# Investigation of Binding Affinity between Potential Antiviral Agents and PB2 Protein of Influenza A: Non-equilibrium Molecular Dynamics Simulation Approach

**DOI:** 10.7150/ijms.46231

**Published:** 2020-07-25

**Authors:** Tri Pham, Hoang Linh Nguyen, Tuyn Phan-Toai, Hung Nguyen

**Affiliations:** 1 Institute for Computational Science and Technology, Ho Chi Minh City, Vietnam.; 2 VNUHCM-University of Technology, Ho Chi Minh City, Vietnam.

**Keywords:** Azaindole (4 and 16), hydroxymethyl azaindole (12), protein_PB2, SMD, MM-PBSA

## Abstract

The PB2 protein of the influenza virus RNA polymerase is a major virulence determinant of influenza viruses. It binds to the cap structure at the 5' end of host mRNA to generate short capped RNA fragments that are used as primers for viral transcription named cap-snatching. A large number of the compounds were shown to bind the minimal cap-binding domain of PB2 to inhibit the cap-snatching machinery. However, their binding in the context of an extended form of the PB2 protein has remained elusive. A previous study reported some promising compounds including azaindole and hydroxymethyl azaindole, which were analyzed here to predict binding affinity to PB2 protein using the steered molecular dynamics (SMD) and molecular mechanics Poisson-Boltzmann surface area (MM-PBSA) methods. The results show that the rupture force (F_max_) value of three complexes is in agreement with the binding free energy value (ΔG_bind_) estimated by the MM-PBSA method, whereas for the non-equilibrium pulling work (W_pull_) value a small difference between A_PB2-4 and A_PB2-12 was observed. The binding affinity results indicate the A_PB2-12 complex is more favorable than the A_PB2-4 and A_PB2-16 complexes, which means the inhibitor (12) has the potential to be further developed as anti-influenza agents in the treatment of influenza A.

## Introduction

Both seasonal and pandemic influenza have been causing severe illness and death for both humans and farm animals 1. The virus variants have emerged and posed a constant threat to human health. Changes in viral genes often result in viral evolutionary advantages, such as the degree of virulence and the more efficient replication and transmissibility of the virus 2.

There has been four main influenza A pandemics, including the H1N1 Spanish flu (1918), the H2N2 Asian flu (1957), the H3N2 Hong Kong flu (1968), and the H1N1 swine flu (2009) 3,4. These have been examples of fast transmission of animal influenza viruses to humans 5. Although vaccination could prevent influenza for 70-90% of healthy adults 6,7, it is only effective against a limited range of strains and it practically has no effect against new and potentially pandemic strains 8-10. Therefore, the development of effective drugs against influenza is currently considered as a high priority by different governmental and international agencies, especially by pharmaceutical industries at the worldwide scale.

The influenza virus is an enveloped virus, in which the outer layer is a lipid membrane, which is taken from the host cell where the virus multiplies 11,12. A glycoprotein is a type of protein molecule linked to sugar. When it is located in a cell membrane, it helps to identify and communicate with the cell. The cell uses the glycoproteins embedded in the plasma membrane to get the oligosaccharides on the outside of the cell, which is typically decorated with different oligosaccharides such as hemagglutinin (HA) 13,14 and neuraminidase (NA) 15. The NA protein is actually the target of the antiviral drugs Relenza and Tamiflu 16,17. Additionally, the M2 protein is also a membrane protein and is a target of the antiviral Adamantanes (including Amantadine and Rimantadine) 18,19. However, the long-term effectiveness of these drugs is a matter of great concern due to the emergence of drug-resistant strains of the virus. Thus, there is an urgent need for new agents to prevent and treat the influenza virus infection, especially in high-risk groups and during pandemic influenza.

Some previous studies showed that some small molecule inhibitors were able to make novel targets in the influenza life cycle against 20-30. The genome of the influenza virus includes eight different ribonucleoprotein complexes that enclose eight viral genomic RNA segments. Similarly to other negative-stranded RNA viruses, the viral RNA polymerase of influenza virus is always packaged in the infectious virion as a complex with the nucleoprotein 31-34. The RNA-dependent RNA polymerase of influenza virus is composed of the PA, PB1, and PB2 subunits. A heterotrimeric polymerase is required for RNA transcription and replication that take place in the nucleus during influenza virus infection 33.

The influenza virus is based on a cap-snatching mechanism to accomplish its transcription 35. Following the entrance of the ribonucleoprotein complexes into the nucleus, the 5' cap of the host pre-mRNA in the nucleus is captured by the cap-binding domain of PB2 36. Together with 10-13 nucleotides downstream, the 5' cap is subsequently cleaved off by the N-terminal cap-dependent endonuclease of PA 37. This 5'-capped oligonucleotide is used as the primer for initiation of the viral transcription by PB1 38. This process is called as a “cap-snatching” mechanism, which allows the endonuclease to cleave the 5' caps from host RNAs, and then the latter act as transcription primers 39. Clearly, the PB2 subunit is linked to the initiation of viral transcription and is known as a cap-binding protein 40-42. According to recent studies, a cap-primer-dependent *in vitro* RNA synthesis is affected by the PB2 gene and, therefore, a series of *in vitro* inhibitors has supported such role for PB2 43. monoclonal antibodies specific for the PB2 subunit have interfered with the initiation step of mRNA-primed transcription *in vitro* 44, and antibodies monospecific for the C terminus of PB2 have inhibited cap snatching and cap-dependent transcription *in vitro* but not cap-binding 45. Moreover, antibodies directed to the region from positions 300 to 550 in PB2 inhibited cap snatching and partially affected cap recognition 46,47. However, the activities of both transcription and cap-dependent endonuclease have required the presence of all three subunits of the polymerase and the RNA template 48, 49.

To elucidate some crucial molecular determinants for the interaction of some inhibitors with PB2 protein of influenza A (protein_PB2), the binding affinity of the azaindole (4&16) and hydroxymethyl azaindole (12) for PB2 protein was predicted. For this purpose, the different theoretical methods including steered molecular dynamics (SMD) 50-52 and Molecular Mechanics Poisson-Boltzmann Surface Area (MM-PBSA) 53,54 were used to compute the binding affinities of these inhibitors for protein_PB2.

## Materials and Method

### Preparing the structures

**Figure 1:**
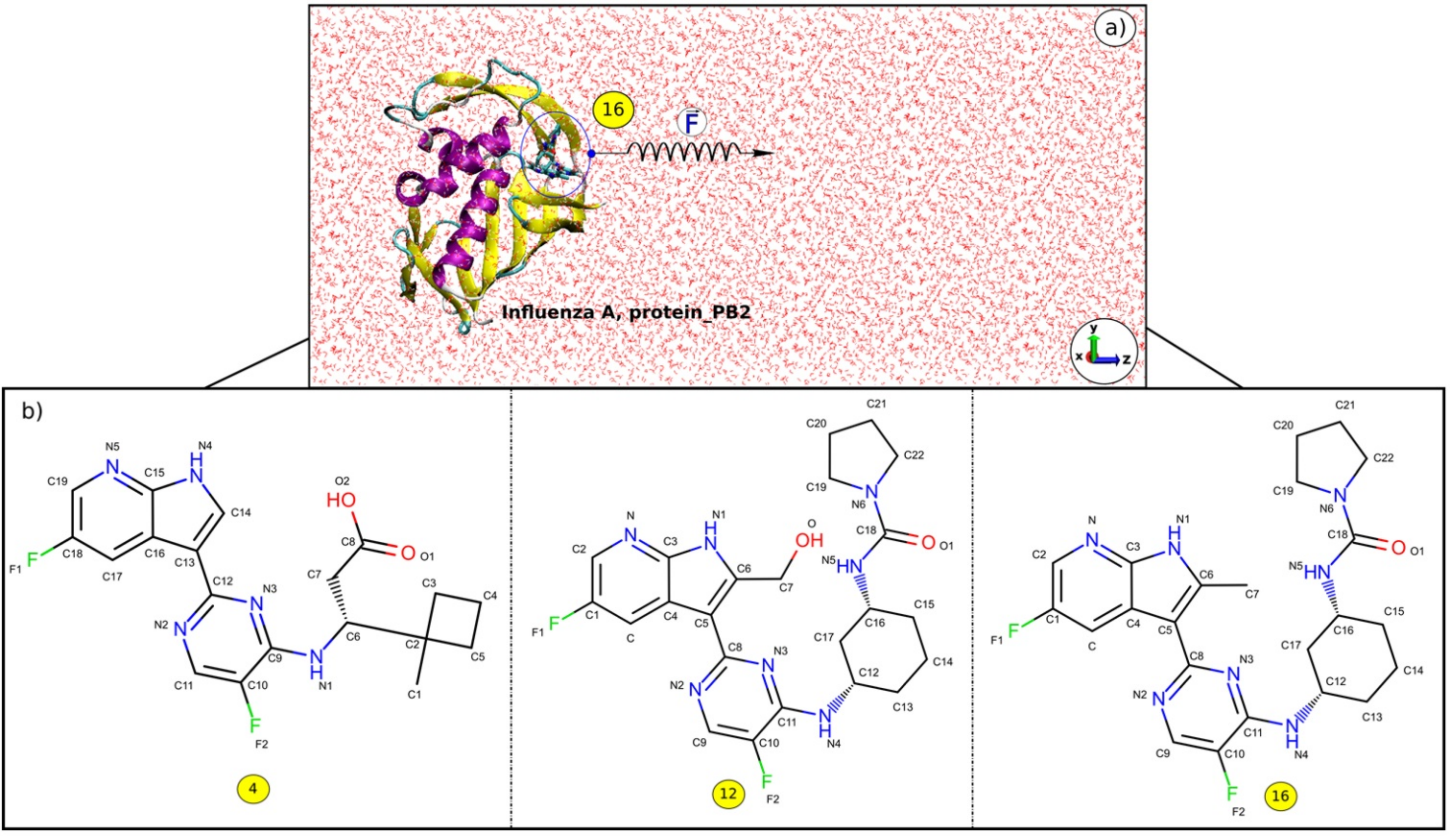
**a**) z-direction of the inhibitor (16) exiting the binding pocket of protein_PB2, and **b**) 2D structures of the inhibitors (4), (12), and (16).

The 3D structures of the complexes were taken from Protein Data Bank with PDB ID: 5JUN (A_PB2-4) 55, 5BUH (A_PB2-12) and 5F79 (A_PB2-16) 56. The 2D structures of the inhibitors (4), (12) and (16) are shown in **Figure 1**. The inhibitor topologies and coordinate files were generated by using Swiss Param 57.

### Molecular dynamics simulations

#### Molecular dynamics simulation

The simulation processes of complexes were conducted by using CHARMM 27 force field 58 implemented in the GROMACS 5.1.2 package 58 at absolute temperature 300 K. The TIP3P water model 60 was used in all simulation systems. All distance bonds within the proteins were constrained by the Linear Constraint Solver (LINCS) algorithm 61. The electrostatic and van der Waals interactions were used to depict non-bonded interactions, with the non-bonded interaction pair-list being updated every 10 fs using a cutoff of 1.4 nm. The Particle Mesh Ewald truncation method 62 was used to treat the long-range electrostatic interactions. From these structures, short 2 ns MD simulations were performed in the NVT ensemble, which were followed by 3 ns NPT simulation. The leap-frog algorithm 63 was used to integrate the equations of motion with the time step set to 2 fs for the MD simulations.

#### Steered molecular dynamics (SMD) simulation

##### Choosing a pathway

Caver 3.0 64 package was used to determine the pulling pathway through the widest tunnel as this minimizes the occurrence of collisions between the inhibitor and protein_PB2 during the simulation. Then the Caver 3.0 and PyMOL 65 packages were employed to rotate the protein_PB2 in such a way that the inhibitor unbinding pathway is along the z-axis (**Figure 1**).

##### Preparing Steered Molecular Dynamics (SMD) simulation

In the Steered Molecular Dynamics (SMD) simulation 50-52, each of the inhibitor-protein_PB2 complexes was placed in a triclinic box of 6nm × 6nm × 14 nm to have enough space to pull the inhibitor out of the binding site. The three-dimensional coordinates of the center of the complex were 3nm × 3nm × 3 nm. The complexes were immersed in a salt solution with a concentration of 0.15 M of sodium and chloride to neutralize the total charge.

The pulling force is measured according to the following equation:


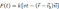
(1)

where 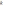 is the force constant, 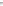 is the pulling velocity, 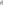 is the pulling direction normal, 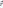 and 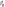 are the positions of protein at time 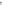 and initial time, respectively. During the simulations, the spring constant 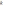 value was set to 600 kJ/ (mol.nm^2^) (approximately 1020 pN/nm), which is a typical value used in atomic force microscope (AFM) experiments 66. The complete dissociation of the inhibitor from the binding pocket of protein_PB2 was reached during 500 ps for three complexes with pulling velocity set at 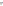 = 0.005 nm/ps.

In order to calculate the relative binding affinities of the complexes using the SMD simulation, the non-equilibrium pulling work profile was used to evaluate a scoring function to rank the binding affinities between the inhibitor and protein. The non-equilibrium pulling work 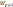 is approximately defined as follows:


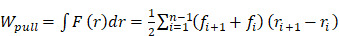
(2)

where 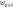
is the non-equilibrium pulling work of external force 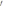, and 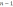 is the number of steps in the SMD simulation, 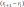 is the inhibitor displacement in step* i*, here the centers of mass (COM) of the inhibitors were employed to measure the inhibitor displacement.

### MM-PBSA free energy calculations

To estimate binding free energy between the inhibitors and protein_PB2, each snapshot extracted from the equilibrium MD simulation was used to estimate the binding free energy using the MM-PBSA method. The binding free energy of the inhibitor-protein is computed by this approach as follows 53,54:


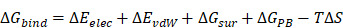
(3)

where 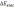 and 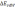 are contributions of electrostatic and vdW energies, respectively 67. 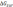 and 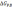 are non-polar and polar solvation energies 68. Here, 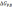 derived from the electrostatic potential between solute and solvent was determined using the continuum solvent approximation 69. It is the change of electrostatic energy from transferring solute in a continuum medium, from a low solute dielectric constant (ε = 2) to a higher one with water without salt (ε = 78.45). Using a grid spacing of 0.1 Å, the APBS package 70 was implemented for numerical solution of the corresponding linear Poisson-Boltzmann equation. The nonpolar solvation term 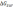 was approximated as linearly dependent on the solvent accessible surface area (SASA), derived from Shrake-Rupley numerical method 71 integrated in the APBS package. 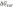 = γSASA + β, where γ = 0.0072 kcal/mol.Å^2^ and β = 0 72. The entropic contribution 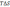 is determined using the normal mode approximation 73.

### Measures used in data analysis

The contact networks between the inhibitors and protein_PB2 were determined by using the LigPlot and PyMOL packages 65,74. The root mean square deviation (RMSD), the number of hydrogen bond (H-bond) and the number of contacts (NC) were calculated by the “gmx_mpi hbond” and “gmx_mpi mindist” tools in the GROMACS package. The standard errors of the mean (E) are approximately estimated as follows:


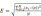
(4)

Where N is the number of snapshots, x_i_ is a quantity at each snapshot, and 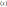 is the average value of x_i_.

## Results

### The contact network and stability of the complexes

**Figure 2:**
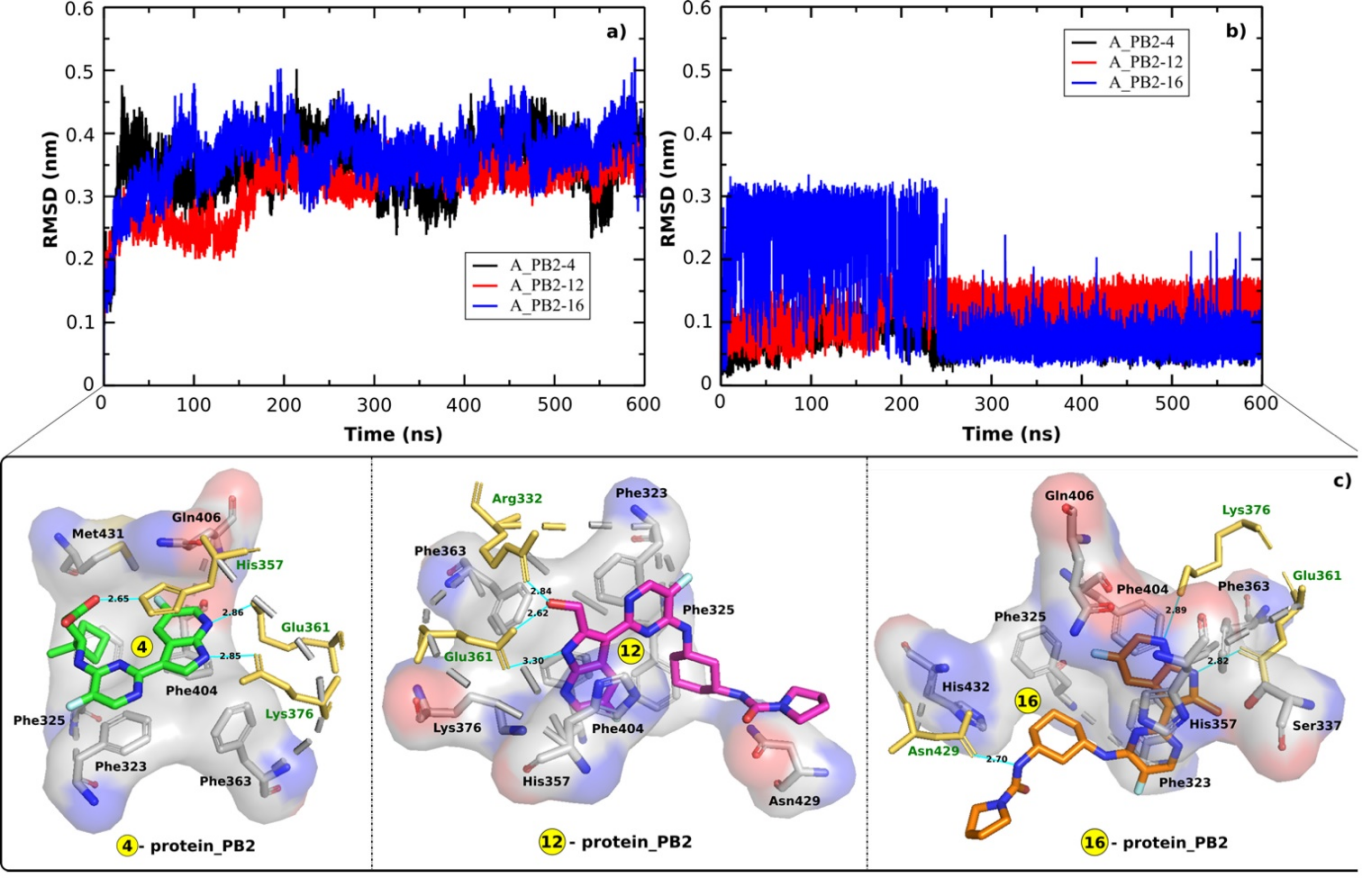
RMSD time profiles for **a)** the backbone of protein_PB2 in the three complexes and **b)** the heavy atoms of three inhibitors. **c**) the contact network between three inhibitors and protein_PB2. Residues shown in yellow form H-bonds (cyan lines) with the ligands.

In order to indicate the stability of the systems including A_PB2-4, A_PB2-12, and A_PB2-16, the RMSD time profiles calculated for protein_PB2's backbone and the inhibitor's heavy atoms are shown in **Figure 2A & B** for every analyzed system. The RMSD values reveal that the three systems reached equilibrium after 250 ns when the RMSD values start fluctuating around about 0.3 nm and 0.45 nm for the backbone of protein_PB2 and around 0.1 nm for the inhibitor's heavy atoms in all cases. The snapshots collected from the last 100 ns of MD simulations were used to calculate the binding free energy through the MM-PBSA method.

The contact network has an important role in stabilizing energetically favored inhibitors. **Figure 2C** displays the contact network between three inhibitors and protein_PB2. By analyzing the interfaces we found the following interactions:

- Both Glu361 and Lys376 form H-bonds with the inhibitors (4) and (16), while only Glu361 forms a H-bond with the inhibitor (12). The Glu361 residue seems to drive the interactions of the inhibitors-protein_PB2 systems. There are three residues forming H-bonds with the inhibitor (4) (Glu361, Lys376, and His357) and the inhibitor (16) (Glu361, Lys376, and Asn429), while only two residues (Glu361 and Arg332) establish H-bonds with the inhibitor (12).
- Most of the H-bonds formed at the interfaces of the three complexes involve side chains of charged (Arg332, His357, Glu361, Lys376) residues, with Asn429 being the only neutral residue whose side chain forms H-bonds with one of the compounds (16). The polar interaction energy can make a large contribution in estimating the binding affinity between the inhibitors and protein_PB2.

The H-bond between the inhibitors and protein_PB2 has an important role in the binding affinity of the complexes. Therefore, the average H-bond occupancies (during the last 100 ns of the MD simulations) were also calculated to determine the contribution of each residue at the interfaces in each complex. The results indicate that the Phe323, Phe325, His357, Glu361, Phe363, Lys376, and Phe404 residues are involved in the formation of a H-bond network in the three complexes (**Table 1**). Most of the residues forming the contact network with three inhibitors are the Phe aromatic residues. That means the Phe residues can have a key role in interactions with the inhibitors and lead to the change of the binding affinity of the inhibitors for protein_PB2.

**Figure 3:**
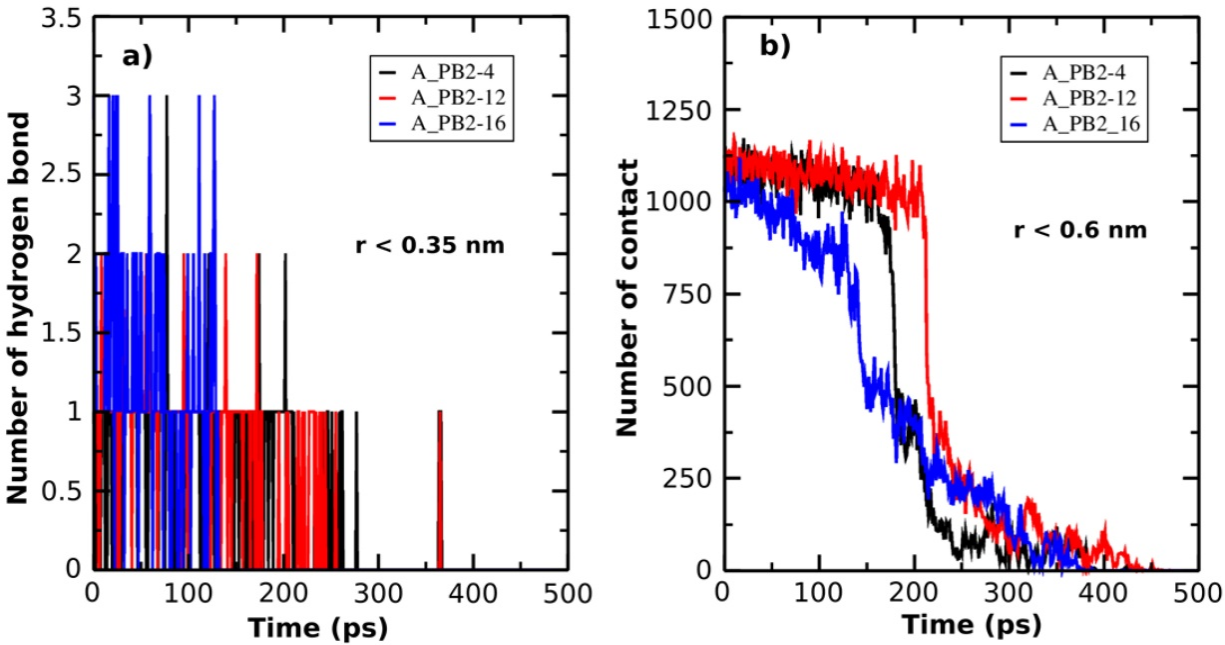
The number of H-bonds (**a**) and the number of NC (**b**) formed between protein_PB2 and the inhibitors are presented as a function of SMD simulation time.

Additionally, in order to describe the unbinding process of the inhibitor-protein_PB2 complexes, the H-bond (r < 0.35nm) and the NC (r < 0.6nm) are determined as a function of simulation time (**Figure 3A & B**). At the bound state, there are three H-bonds formed between inhibitors (4) and (16) and protein_PB2, while there are only 2 H-bonds between inhibitor (12) and protein_PB2. At the unbound state, the number of H-bonds decreases to 0 for all three complexes. Likewise, the NC value also helps follow the unbinding process of the inhibitor-protein_PB2 complexes. There are roughly 1125 NCs formed between the three inhibitors and protein_PB2 at the bound state, which decrease to 0 NCs after 400 ps for inhibitor (4), after 425 ps for inhibitor (16) and after 450 ps for inhibitor (12) at the unbound state. Although the NC and the number of H-bonds of the A_PB2-16 complex are greater than those of the A_PB2-4 and A_PB2-12 complexes, the NC and the number of H-bonds of the A_PB2-16 complex reach 0 faster than those of the A_PB2-4 and A_PB2-12 complexes.

### Binding affinity between the inhibitors and protein_PB2

Binding affinity is widely used to evaluate and rank order strength of biomolecular interactions in biological systems. In this research, to make a full understanding of the binding mechanism of these inhibitors to protein_PB2, we estimated the binding affinity of the azaindole (4&16) and hydroxymethyl azaindole (12) to protein_PB2.

**Figure 4:**
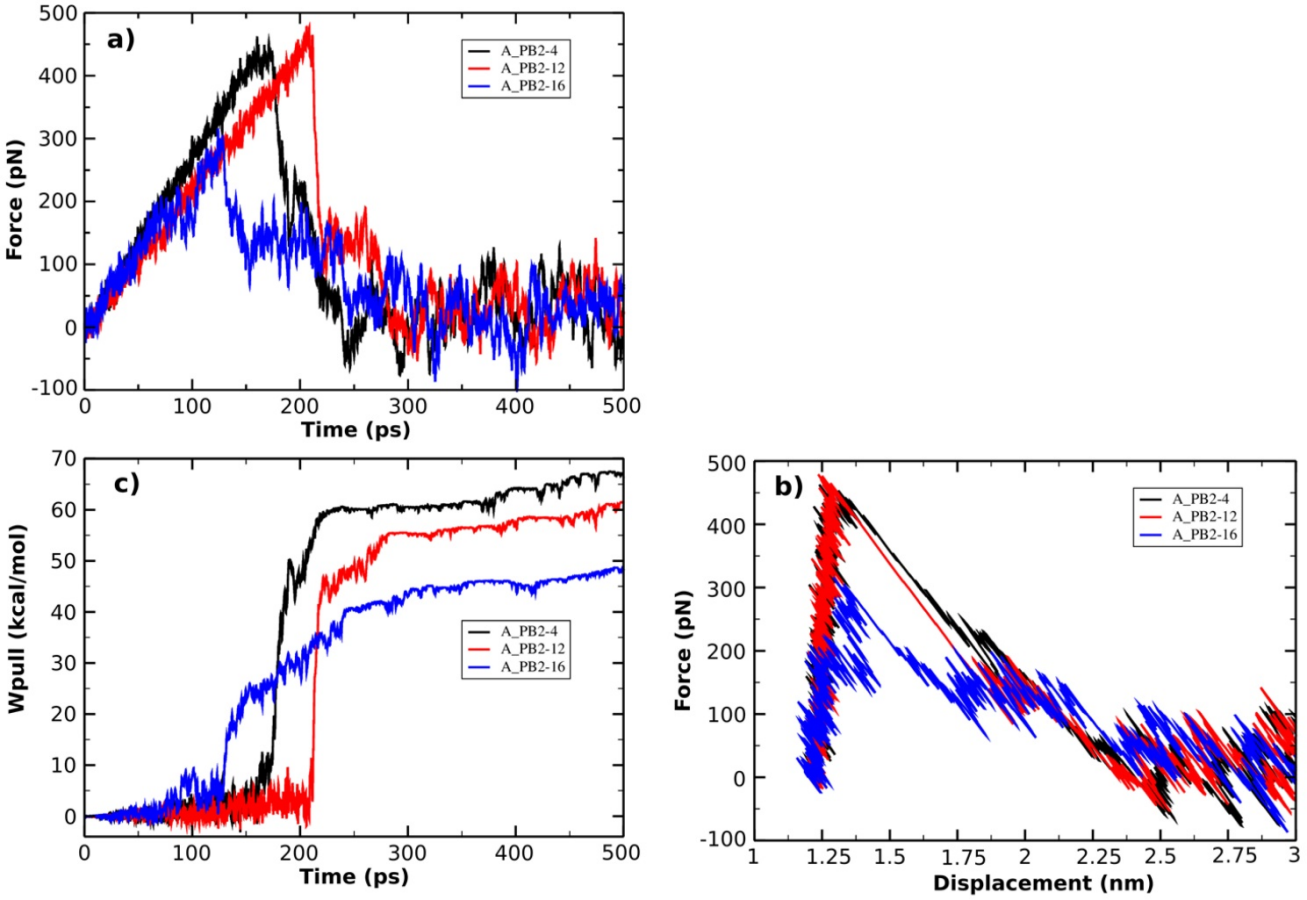
(**a**) Force vs time and (**b**) force vs displacement profiles. (**c**) Pulling work-time profile.

The SMD is used as a method to study the unbinding process of small molecules (the inhibitors) from a large molecule (protein_PB2) and is capable of rank the compounds binding a same target protein according to their relative binding affinities 75. The pulling force profile is started as a function of time (shown in **Figure 4A**). The F_max_ has an important role to indicate the dissociation between protein_PB2 and the inhibitors. Here the pulling force profile is able to show the division of two consecutive stages: During the first stage, the pulling force continuously increased until the inhibitors started to dissociate from protein_PB2, and the external force reached the F_max_ value when the H-bonds are broken. During the second stage, the pulling force started to decrease as the inhibitors are exiting the binding pocket of protein_PB2. In more detail, the largest pulling force of the A_PB2-12 complex (F_max_ = 477.50 pN, *t=*220 ps) is larger than those of the A_PB2-4 complex (F_max_ = 462.42 pN, *t=*170 ps) and the A_PB2-16 complex (F_max_ = 315.86 pN, *t=*125 ps), respectively. Overall, the variation of pulling force profiles among the inhibitor-protein_PB2 complexes could be attributed to the binding site of protein_PB2. Moreover, the evolution of the steering force during the inhibitor displacement first showed a linear behavior which then became nonlinear before the F_max_ value was reached (seen from **Figure 4B**). At this point, the inhibitor is still located in the binding pocket. After obtaining the F_max_ value, the force value decreased steeply and fluctuated around zero value (beginning at 2.25 nm), when the inhibitor detached from the protein_PB2 along the pulling direction.

The non-equilibrium pulling work (W_pull_) is also used to predict the relative binding affinity of protein_PB2-inhibitor systems (eq. 2). As seen from **Figure 4C**, the W_pull_ rapidly increased as the pulling force does, until the inhibitors come out from the binding pocket of protein_PB2. It reached a stable value when the inhibitors lost their non-bonded contacts with protein_PB2 (corresponding to 50 kcal/mol for (16), 60 kcal/mol for (12) and 65 kcal/mol for (4)). However, note that small fluctuations occur, which can be neglected. All three systems reach a stable state after 250 ps.

In this work, the MM-PBSA method is also used to estimate the binding free energies and makes possible to indicate the contribution of each energy component to the inhibitor's potency. As seen in **Table 2**, the contribution of different energy components to the ΔG_bind_ indicates that the electrostatic energy (ΔE_elec_) has a more important role than the vdW energy (ΔE_vdW_). The changes in the nonpolar solvation energy (ΔG_sur_) values significantly contributed to the difference of the ΔG_bind_ among the complexes. The entropy (-TΔS) of the A_PB2-12 complex is larger than those of the A_PB2-4 and A_PB2-16 complexes. The loss of polar solvation energy (ΔG_PB_) is compensated by the remaining components of the ΔG_bind_ in these systems.

Specifically, the ΔE_elec_ obtained from the complex formation compensates the loss in ΔG_PB_. Here, the ΔE_elec_ of the A_PB2-12 complex (-16.43 kcal/mol) is more negative than those of both remaining complexes (-7.63 kcal/mol for the A_PB2-4 complex, and -7.71 kcal/mol for the A_PB2-16 complex). The ΔG_vdW_ values of the three complexes (ranged from -1.86 to -3.60 kcal/mol) do not lead to the difference of their ΔG_bind_ values. Conversely, the ΔG_sur_ values of these complexes significantly contribute to the difference of their ΔG_bind_ values (ranged from -6.95 to -4.41 kcal/mol). The entropy contribution (-TΔS) of the A_PB2-12 complex (7.36 kcal/mol) is greater than those of the A_PB2-4 and A_PB2-16 complexes (0.52 kcal/mol for the A_PB2-4 complex, and 0.49 kcal/mol for the A_PB2-16 complex). Clearly, the OH group of inhibitor (12) is not only yielded to a more negative value of electrostatic energy but also contributed to making a fairly large entropic energy. Here, the -TΔS value of the A_PB2-12 complex contributed appreciably to the change of the binding free energy while this energy component has a negligible impact on the binding free energy to two remaining complexes. Finally, the ΔG_bind_ of the A_PB2-12 complex (-12.70 kcal/mol) is lower than that of both remaining complexes (-10.04 kcal/mol for the A_PB2-4 complex, and -6.44 kcal/mol for the A_PB2-16 complex). The results indicate that the affinity of inhibitor (12) bound for protein_PB2 is stronger than that of inhibitors (4) and (16). Moreover, our calculations reveal that the F_max_ of three complexes is in good agreement with the ΔG_bind_ value, while the W_pull_ value has a small difference between A_PB2-4 and A_PB2-12. In short, the inhibitor (12) has the potential to be further developed as anti-influenza agents in the treatment of influenza A.

## Concluding Remarks

In the present theoretical study, we applied the SMD and MM-PBSA methods to predict the binding affinity of three inhibitors for protein_PB2. A number of interesting results emerged from our work, which can be summarized as follows:

- The results from the MM-PBSA method showed that the electrostatics (ΔE_elec_) interaction plays a more important role than the van der Waals (ΔE_vdW_) component in contributing to the binding free energy value of all three complexes. Additionally, the entropy (-TΔS) of the A_PB2-12 complex has a large detrimental impact on the binding free energy while it makes no significant contribution to the binding free energies of the A_PB2-4 and A_PB2-16 complexes.
- The F_max_ value of three complexes is in agreement with the ΔG_bind_ value, while the W_pull_ value a small difference between the A_PB2-4 and A_PB2-12 complexes was observed. The binding affinities showed that the affinity of inhibitor (12) for protein_PB2 is stronger than that of inhibitors (4) and (16).

## Supplementary Material

Supplementary parameterization scheme.

## Figures and Tables

**Table 1:** Average occupancies of H-bonds between the inhibitors and protein_PB2 in the three A_PB2-4, A_PB2-12, and A_PB2-16 complexes, determined from the last 100 ns of the MD simulations

Residue	Inhibitor (4)	Inhibitor (12)	Inhibitor (16)
Phe323 (O)	0.1873 (N4)	0.0055 (N1)	0.0044 (F2)
Phe325 (O)	0.0431 (N4)	0.1101 (F2)	0.0112 (N5)
Arg332 (N)		0.4688 (N2, F1, O)	
Ser337 (O)			0.0019 (N)
His357 (N)	0.6012 (O1, O2)	0.2155 (N2, O, F1, F2)	0.0377 (O1, F2)
Glu361 (O)	0.5202 (N5)	0.8033 (N1, O)	0.5010 (N1)
Phe363(O)	0.0323(N4)	0.0795 (N1)	0.2188 (N1)
Lys376 (N)	0.4432 (N4)	0.1113 (N, O)	0.6212 (N)
Phe404 (O)	0.2261 (F1)	0.2278 (O)	0.0220 (N)
Gln406 (N)	0.0110 (N5)		0.0060 (N, N2, F1, F2)
Asn429 (N)		0.2032 (N6, O1, F1)	0.5721 (F1, N5)
Met431 (O)	0.0032 (N4)		
His432 (N)			0.0731 (O1)

**Table 2:** Binding affinity values of three inhibitors for protein_PB2, estimated from the MM-PBSA method and SMD simulations

	ΔE_elec_ (kcal/mol)	ΔE_vdW_ (kcal/mol)	ΔG_sur_ (kcal/mol)	ΔG_PB_ (kcal/mol)	-TΔS (kcal/mol)	ΔG_bind_ (kcal/mol)	F_max_ (pN)	W_pull_ (kcal/mol)
(4)	-7.63±1.77	-2.17±0.25	-6.95±1.05	6.19±1.31	0.52±0.02	-10.04±1.63	462.42±47.12	77.13±8.25
(12)	-16.43±3.15	-3.60±0.11	-6.77±2.02	6.74±1.73	7.36±1.24	-12.70±1.41	477.50±51.61	72.04±7.77
(16)	-7.71±1.14	-1.86±0.05	-4.41±0.93	7.05±1.79	0.49±0.02	-6.44±1.12	315.86±39.32	55.12±6.75
