# Supplementary material for: Investigation of Binding Affinity between Potential Antiviral Agents and PB2 Protein of Influenza A: Non-equilibrium Molecular Dynamics Simulation Approach
Source: Int J Med Sci. 2020 Jul 25;17(13):2031–9. doi: 10.7150/ijms.46231 (PMC7415388; doi:10.7150/ijms.46231)
Supplement: Supplementary file 1 — Supplementary parameterization scheme. [file ijmsv17p2031s1.pdf]

Investigation of Binding Affinity between Potential Antiviral Agents and PB2 Protein of Influenza A: Non-equilibrium Molecular Dynamics Simulation Approach

Tri Pham[1,2], Hoang Linh Nguyen[1,2], Tuyn Phan-Toai[1], and Hung Nguyen[1,*]

[1]*Institute for Computational Science and Technology, Ho Chi Minh City, Vietnam.*

[2]*VNUHCM-University of Technology, Ho Chi Minh City, Vietnam.*

[*]*Email: hung.nv@icst.org.vn*

SUPPORTING INFORMATION

The parameterization scheme used for three compounds is shown below:

1) The inhibitor (4):

```
[ atomtypes ]
; name at.num  mass   charge  ptype   sigma          epsilon
CB      6   12.0110  0.0  A        0.355005    0.292880
NPYD    7   14.0067  0.0  A        0.329632    0.836800
C5B     6   12.0110  0.0  A        0.363487    0.209200
C5A     6   12.0110  0.0  A        0.363487    0.209200
NPYL    7   14.0067  0.0  A        0.306469    0.376560
F       9   18.9984  0.0  A        0.290433    0.564840
NM      7   14.0067  0.0  A        0.329632    0.836800
CR      6   12.0110  0.0  A        0.387541    0.230120
CO2M    6   12.0110  0.0  A        0.356359    0.292880
O2CM    8   15.9994  0.0  A        0.302905    0.502080
CR4R    6   12.0110  0.0  A        0.333196    0.589944
HCMM    1    1.0079  0.0  A        0.235197    0.092048
HNR     1    1.0079  0.0  A        0.040001    0.192464


[ pairtypes ]
; i    j    func     sigma1-4      epsilon1-4 ; THESE ARE 1-4 INTERACTIONS
CR     CB     1     0.346773    0.110698
CR     NPYD   1     0.334087    0.187114
CR     C5B    1     0.351014    0.093557
CR     C5A    1     0.351014    0.093557
CR     NPYL   1     0.322505    0.125520
CR     F      1     0.314487    0.153730
CR     NM     1     0.334087    0.187114
CR     CR     1     0.338541    0.041840
CR     CO2M   1     0.347450    0.110698
CR     O2CM   1     0.320723    0.144938
CR     CR4R   1     0.335869    0.157109
CR     HCMM   1     0.286869    0.062059
CR     HNR    1     0.189271    0.089737

[ moleculetype ]
; Name nrexcl
```

4 3

```
[ atoms ]
; nr type resnr resid atom cgnr charge mass
   1 CB   1  LIG C       1  0.1900  12.0110
   2 CB   1  LIG C1      2  0.1600  12.0110
   3 NPYD 1  LIG N       3 -0.6200  14.0067
   4 CB   1  LIG C2      4  0.6740  12.0110
   5 NPYD 1  LIG N1      5 -0.6200  14.0067
   6 CB   1  LIG C3      6  0.0580  12.0110
   7 C5B  1  LIG C12     7 -0.0540  12.0110
   8 C5B  1  LIG C13     8  0.0000  12.0110
   9 CB   1  LIG C14     9 -0.1500  12.0110
  10 CB   1  LIG C15    10  0.1900  12.0110
  11 CB   1  LIG C16    11  0.1600  12.0110
  12 NPYD 1  LIG N3     12 -0.5670  14.0067
  13 C5A  1  LIG C17    13  0.1054  12.0110
  14 NPYL 1  LIG N4     14  0.0332  14.0067
  15 C5A  1  LIG C18    15 -0.3016  12.0110
  16 F    1  LIG F      16 -0.1900  18.9984
  17 NM   1  LIG N2     17 -0.6980  14.0067
  18 CR   1  LIG C4     18 -0.0500  12.0110
  19 CR   1  LIG C5     19 -0.1060  12.0110
  20 CO2M 1  LIG C6     20  0.9060  12.0110
  21 O2CM 1  LIG O      21 -0.9000  15.9994
  22 O2CM 1  LIG O1     22 -0.9000  15.9994
  23 CR4R 1  LIG C7     23  0.0000  12.0110
  24 CR4R 1  LIG C8     24  0.0000  12.0110
  25 CR4R 1  LIG C9     25  0.0000  12.0110
  26 CR4R 1  LIG C10    26  0.0000  12.0110
  27 CR   1  LIG C11    27  0.0000  12.0110
  28 F    1  LIG F1     28 -0.1900  18.9984
  29 HCMM 1  LIG H      29  0.1500   1.0079
  30 HCMM 1  LIG H1     30  0.0000   1.0079
  31 HCMM 1  LIG H2     31  0.0000   1.0079
  32 HCMM 1  LIG H3     32  0.0000   1.0079
  33 HCMM 1  LIG H4     33  0.0000   1.0079
  34 HCMM 1  LIG H5     34  0.0000   1.0079
  35 HCMM 1  LIG H6     35  0.0000   1.0079
  36 HCMM 1  LIG H7     36  0.0000   1.0079
  37 HCMM 1  LIG H8     37  0.0000   1.0079
  38 HCMM 1  LIG H9     38  0.0000   1.0079
  39 HCMM 1  LIG H10    39  0.0000   1.0079
  40 HCMM 1  LIG H11    40  0.0000   1.0079
  41 HCMM 1  LIG H12    41  0.0000   1.0079
  42 HCMM 1  LIG H13    42  0.1500   1.0079
  43 HCMM 1  LIG H14    43  0.1500   1.0079
  44 HNR  1  LIG H15    44  0.2700   1.0079
  45 HCMM 1  LIG H16    45  0.1500   1.0079

[ bonds ]
; ai aj fu b0 kb, b0 kb
 20  21 1 0.12610  587519.8  0.12610  587519.8
 20  22 1 0.12610  587519.8  0.12610  587519.8
 44  14 1 0.10120  428294.3  0.10120  428294.3
 14  13 1 0.13640  379454.5  0.13640  379454.5
 14  15 1 0.13640  379454.5  0.13640  379454.5
```

```
 12  13 1 0.13300  439556.0  0.13300  439556.0
 12  11 1 0.13330  345489.6  0.13330  345489.6
 43  11 1 0.10840  319534.6  0.10840  319534.6
 13   8 1 0.13770  428655.8  0.13770  428655.8
 45  15 1 0.10800  333084.1  0.10800  333084.1
 11  10 1 0.13740  335613.7  0.13740  335613.7
 15   7 1 0.13770  428655.8  0.13770  428655.8
  8   7 1 0.14180  259734.4  0.14180  259734.4
  8   9 1 0.13790  371023.7  0.13790  371023.7
  7   4 1 0.13790  371023.7  0.13790  371023.7
 10   9 1 0.13740  335613.7  0.13740  335613.7
 10  28 1 0.13420  392101.0  0.13420  392101.0
  9  42 1 0.10840  319534.6  0.10840  319534.6
  3   4 1 0.13330  345489.6  0.13330  345489.6
  3   2 1 0.13330  345489.6  0.13330  345489.6
  4   5 1 0.13330  345489.6  0.13330  345489.6
  5   6 1 0.13330  345489.6  0.13330  345489.6
 29   2 1 0.10840  319534.6  0.10840  319534.6
  2   1 1 0.13740  335613.7  0.13740  335613.7
 20  19 1 0.15100  230648.0  0.15100  230648.0
  6   1 1 0.13740  335613.7  0.13740  335613.7
  6  17 1 0.13350  429799.7  0.13350  429799.7
 30  18 1 0.10930  287014.9  0.10930  287014.9
  1  16 1 0.13420  392101.0  0.13420  392101.0
 35  25 1 0.10930  292193.8  0.10930  292193.8
 18  19 1 0.15080  256422.3  0.15080  256422.3
 18  17 1 0.14440  268346.7  0.14440  268346.7
 18  23 1 0.15040  280029.3  0.15040  280029.3
 19  32 1 0.10930  287014.9  0.10930  287014.9
 19  31 1 0.10930  287014.9  0.10930  287014.9
 38  26 1 0.10930  292193.8  0.10930  292193.8
 33  24 1 0.10930  292193.8  0.10930  292193.8
 25  36 1 0.10930  292193.8  0.10930  292193.8
 25  26 1 0.15260  220590.5  0.15260  220590.5
 25  24 1 0.15260  220590.5  0.15260  220590.5
 26  23 1 0.15260  220590.5  0.15260  220590.5
 26  37 1 0.10930  292193.8  0.10930  292193.8
 24  23 1 0.15260  220590.5  0.15260  220590.5
 24  34 1 0.10930  292193.8  0.10930  292193.8
 23  27 1 0.15040  280029.3  0.15040  280029.3
 40  27 1 0.10930  287014.9  0.10930  287014.9
 27  41 1 0.10930  287014.9  0.10930  287014.9
 27  39 1 0.10930  287014.9  0.10930  287014.9

[ pairs ]
; ai aj fu
  1   4 1
  1  18 1
  2   5 1
  2  17 1
  2   7 1
  3   6 1
  3  16 1
  3   8 1
  3  15 1
  4  29 1
  4  17 1
```

4	9	1
4	13	1
4	14	1
4	45	1
5	8	1
5	15	1
5	16	1
5	18	1
6	29	1
6	7	1
6	19	1
6	23	1
6	30	1
7	10	1
7	42	1
7	12	1
7	44	1
8	45	1
8	11	1
8	28	1
8	44	1
9	15	1
9	12	1
9	14	1
9	43	1
10	13	1
11	42	1
11	14	1
12	28	1
12	15	1
12	44	1
13	42	1
13	43	1
13	45	1
16	29	1
16	17	1
17	20	1
17	31	1
17	32	1
17	24	1
17	26	1
17	27	1
18	21	1
18	22	1
18	25	1
18	33	1
18	34	1
18	37	1
18	38	1
18	39	1
18	40	1
18	41	1
19	24	1
19	26	1
19	27	1
20	23	1
20	30	1

```
 21  31 1
 21  32 1
 22  31 1
 22  32 1
 23  31 1
 23  32 1
 23  35 1
 23  36 1
 24  30 1
 24  37 1
 24  38 1
 24  39 1
 24  40 1
 24  41 1
 25  27 1
 26  30 1
 26  33 1
 26  34 1
 26  39 1
 26  40 1
 26  41 1
 27  30 1
 27  33 1
 27  34 1
 27  37 1
 27  38 1
 28  42 1
 28  43 1
 30  31 1
 30  32 1
 33  35 1
 33  36 1
 34  35 1
 34  36 1
 35  37 1
 35  38 1
 36  37 1
 36  38 1
 44  45 1

[ angles ]
; ai aj ak fu th0 kth ub0 kub th0 kth ub0 kub
  2   1    6 1  119.9770  402.88    119.9770  402.88
  2   1   16 1  118.0650  658.81    118.0650  658.81
  6   1   16 1  118.0650  658.81    118.0650  658.81
  1   2    3 1  126.1390  358.92    126.1390  358.92
  1   2   29 1  120.5710  339.05    120.5710  339.05
  3   2   29 1  115.5880  417.33    115.5880  417.33
  2   3    4 1  115.4060  653.40    115.4060  653.40
  3   4    5 1  128.9380  436.60    128.9380  436.60
  3   4    7 1  128.9380  436.60    128.9380  436.60
  5   4    7 1  128.9380  436.60    128.9380  436.60
  4   5    6 1  115.4060  653.40    115.4060  653.40
  1   6    5 1  126.1390  358.92    126.1390  358.92
  1   6   17 1  124.3840  566.68    124.3840  566.68
  5   6   17 1  118.3490  691.34    118.3490  691.34
  4   7    8 1  128.6730  464.91    128.6730  464.91
```

4	7	15	1	120.0000	602.21	120.0000	602.21
8	7	15	1	108.2390	521.51	108.2390	521.51
7	8	9	1	128.6730	464.91	128.6730	464.91
7	8	13	1	108.2390	521.51	108.2390	521.51
9	8	13	1	120.0000	602.21	120.0000	602.21
8	9	10	1	112.5670	254.73	112.5670	254.73
8	9	42	1	121.4460	314.95	121.4460	314.95
10	9	42	1	120.5710	339.05	120.5710	339.05
9	10	11	1	119.9770	402.88	119.9770	402.88
9	10	28	1	118.0650	658.81	118.0650	658.81
11	10	28	1	118.0650	658.81	118.0650	658.81
10	11	12	1	126.1390	358.92	126.1390	358.92
10	11	43	1	120.5710	339.05	120.5710	339.05
12	11	43	1	115.5880	417.33	115.5880	417.33
11	12	13	1	110.1810	740.72	110.1810	740.72
8	13	12	1	126.5130	548.01	126.5130	548.01
8	13	14	1	107.2550	489.59	107.2550	489.59
12	13	14	1	124.8140	615.46	124.8140	615.46
13	14	15	1	109.5990	693.74	109.5990	693.74
13	14	44	1	127.7700	331.82	127.7700	331.82
15	14	44	1	127.7700	331.82	127.7700	331.82
7	15	14	1	107.2550	489.59	107.2550	489.59
7	15	45	1	131.7210	347.47	131.7210	347.47
14	15	45	1	121.1270	371.56	121.1270	371.56
6	17	18	1	120.0000	602.21	120.0000	602.21
17	18	19	1	109.5000	602.21	109.5000	602.21
17	18	23	1	109.5000	602.21	109.5000	602.21
17	18	30	1	113.0350	394.45	113.0350	394.45
19	18	23	1	108.6590	614.86	108.6590	614.86
19	18	30	1	110.5490	383.00	110.5490	383.00
23	18	30	1	111.0000	425.16	111.0000	425.16
18	19	20	1	98.4220	198.73	98.4220	198.73
18	19	31	1	110.5490	383.00	110.5490	383.00
18	19	32	1	110.5490	383.00	110.5490	383.00
20	19	31	1	108.9040	316.16	108.9040	316.16
20	19	32	1	108.9040	316.16	108.9040	316.16
31	19	32	1	108.8360	310.74	108.8360	310.74
19	20	21	1	114.6890	728.07	114.6890	728.07
19	20	22	1	114.6890	728.07	114.6890	728.07
21	20	22	1	130.6000	711.20	130.6000	711.20
18	23	24	1	113.3130	302.31	113.3130	302.31
18	23	26	1	113.3130	302.31	113.3130	302.31
18	23	27	1	113.1310	567.89	113.1310	567.89
24	23	26	1	108.6440	607.02	108.6440	607.02
24	23	27	1	113.3130	302.31	113.3130	302.31
26	23	27	1	113.3130	302.31	113.3130	302.31
23	24	25	1	108.6440	607.02	108.6440	607.02
23	24	33	1	113.9400	339.65	113.9400	339.65
23	24	34	1	113.9400	339.65	113.9400	339.65
25	24	33	1	113.9400	339.65	113.9400	339.65
25	24	34	1	113.9400	339.65	113.9400	339.65
33	24	34	1	109.1070	264.37	109.1070	264.37
24	25	26	1	108.6440	607.02	108.6440	607.02
24	25	35	1	113.9400	339.65	113.9400	339.65
24	25	36	1	113.9400	339.65	113.9400	339.65
26	25	35	1	113.9400	339.65	113.9400	339.65
26	25	36	1	113.9400	339.65	113.9400	339.65

35	25	36	1	109.1070	264.37	109.1070	264.37
23	26	25	1	108.6440	607.02	108.6440	607.02
23	26	37	1	113.9400	339.65	113.9400	339.65
23	26	38	1	113.9400	339.65	113.9400	339.65
25	26	37	1	113.9400	339.65	113.9400	339.65
25	26	38	1	113.9400	339.65	113.9400	339.65
37	26	38	1	109.1070	264.37	109.1070	264.37
23	27	39	1	111.0000	425.16	111.0000	425.16
23	27	40	1	111.0000	425.16	111.0000	425.16
23	27	41	1	111.0000	425.16	111.0000	425.16
39	27	40	1	108.8360	310.74	108.8360	310.74
39	27	41	1	108.8360	310.74	108.8360	310.74
40	27	41	1	108.8360	310.74	108.8360	310.74

[dihedrals]

; ai	aj	ak	al	fu	phi0	kphi	mult	phi0	kphi	mult
1	2	3	4	9	180.00	14.6440	2	180.00	14.6440	2
1	6	5	4	9	180.00	14.6440	2	180.00	14.6440	2
1	6	17	18	9	180.00	7.5312	2	180.00	7.5312	2
2	1	6	5	9	180.00	14.6440	2	180.00	14.6440	2
2	1	6	17	9	180.00	14.6440	2	180.00	14.6440	2
2	3	4	5	9	180.00	14.6440	2	180.00	14.6440	2
2	3	4	7	9	180.00	14.6440	2	180.00	14.6440	2
3	2	1	6	9	180.00	14.6440	2	180.00	14.6440	2
3	2	1	16	9	180.00	14.6440	2	180.00	14.6440	2
3	4	5	6	9	180.00	14.6440	2	180.00	14.6440	2
3	4	7	8	9	180.00	14.6440	2	180.00	14.6440	2
3	4	7	15	9	180.00	14.6440	2	180.00	14.6440	2
4	3	2	29	9	180.00	14.6440	2	180.00	14.6440	2
4	5	6	17	9	180.00	14.6440	2	180.00	14.6440	2
4	7	8	9	9	180.00	3.7656	2	180.00	3.7656	2
4	7	8	13	9	180.00	3.7656	2	180.00	3.7656	2
5	4	7	8	9	180.00	14.6440	2	180.00	14.6440	2
5	4	7	15	9	180.00	14.6440	2	180.00	14.6440	2
5	6	1	16	9	180.00	14.6440	2	180.00	14.6440	2
5	6	17	18	9	180.00	7.5312	2	180.00	7.5312	2
6	1	2	29	9	180.00	14.6440	2	180.00	14.6440	2
6	5	4	7	9	180.00	14.6440	2	180.00	14.6440	2
6	17	18	19	9	0.00	0.5230	3	0.00	0.5230	3
6	17	18	23	9	0.00	0.5230	3	0.00	0.5230	3
6	17	18	30	9	0.00	0.5230	3	0.00	0.5230	3
7	8	9	10	9	180.00	14.6440	2	180.00	14.6440	2
7	8	9	42	9	180.00	14.6440	2	180.00	14.6440	2
7	8	13	12	9	180.00	14.6440	2	180.00	14.6440	2
7	8	13	14	9	180.00	14.6440	2	180.00	14.6440	2
7	15	14	13	9	180.00	8.3680	2	180.00	8.3680	2
7	15	14	44	9	180.00	8.3680	2	180.00	8.3680	2
8	7	15	14	9	180.00	14.6440	2	180.00	14.6440	2
8	7	15	45	9	180.00	14.6440	2	180.00	14.6440	2
8	9	10	11	9	180.00	14.6440	2	180.00	14.6440	2
8	9	10	28	9	180.00	14.6440	2	180.00	14.6440	2
8	13	12	11	9	180.00	14.6440	2	180.00	14.6440	2
8	13	14	15	9	180.00	8.3680	2	180.00	8.3680	2
8	13	14	44	9	180.00	8.3680	2	180.00	8.3680	2
9	8	7	15	9	180.00	3.7656	2	180.00	3.7656	2
9	8	13	12	9	180.00	14.6440	2	180.00	14.6440	2
9	10	11	12	9	180.00	14.6440	2	180.00	14.6440	2

9	10	11	43	9	180.00	14.6440	2	180.00	14.6440	2
10	9	8	13	9	180.00	14.6440	2	180.00	14.6440	2
10	11	12	13	9	180.00	14.6440	2	180.00	14.6440	2
11	10	9	42	9	180.00	14.6440	2	180.00	14.6440	2
11	12	13	14	9	180.00	14.6440	2	180.00	14.6440	2
12	11	10	28	9	180.00	14.6440	2	180.00	14.6440	2
12	13	14	15	9	180.00	8.3680	2	180.00	8.3680	2
12	13	14	44	9	180.00	8.3680	2	180.00	8.3680	2
13	8	7	15	9	180.00	14.6440	2	180.00	14.6440	2
13	8	9	42	9	180.00	14.6440	2	180.00	14.6440	2
13	12	11	43	9	180.00	14.6440	2	180.00	14.6440	2
13	14	15	45	9	180.00	8.3680	2	180.00	8.3680	2
16	1	2	29	9	180.00	14.6440	2	180.00	14.6440	2
16	1	6	17	9	180.00	14.6440	2	180.00	14.6440	2
17	18	19	20	9	0.00	0.6276	3	0.00	0.6276	3
17	18	19	31	9	0.00	0.6276	3	0.00	0.6276	3
17	18	19	32	9	0.00	0.6276	3	0.00	0.6276	3
17	18	23	24	9	0.00	0.7322	3	0.00	0.7322	3
17	18	23	26	9	0.00	0.7322	3	0.00	0.7322	3
17	18	23	27	9	0.00	0.7322	3	0.00	0.7322	3
18	19	20	21	9	180.00	2.6401	2	180.00	2.6401	2
18	19	20	22	9	180.00	2.6401	2	180.00	2.6401	2
18	23	24	25	9	0.00	-0.1339	1	0.00	-0.1339	1
18	23	24	25	9	180.00	-0.1339	2	180.00	-0.1339	2
18	23	24	25	9	0.00	0.2929	3	0.00	0.2929	3
18	23	24	33	9	0.00	0.1423	1	0.00	0.1423	1
18	23	24	33	9	180.00	0.1715	2	180.00	0.1715	2
18	23	24	33	9	0.00	0.7238	3	0.00	0.7238	3
18	23	24	34	9	0.00	0.1423	1	0.00	0.1423	1
18	23	24	34	9	180.00	0.1715	2	180.00	0.1715	2
18	23	24	34	9	0.00	0.7238	3	0.00	0.7238	3
18	23	26	25	9	0.00	-0.1339	1	0.00	-0.1339	1
18	23	26	25	9	180.00	-0.1339	2	180.00	-0.1339	2
18	23	26	25	9	0.00	0.2929	3	0.00	0.2929	3
18	23	26	37	9	0.00	0.1423	1	0.00	0.1423	1
18	23	26	37	9	180.00	0.1715	2	180.00	0.1715	2
18	23	26	37	9	0.00	0.7238	3	0.00	0.7238	3
18	23	26	38	9	0.00	0.1423	1	0.00	0.1423	1
18	23	26	38	9	180.00	0.1715	2	180.00	0.1715	2
18	23	26	38	9	0.00	0.7238	3	0.00	0.7238	3
18	23	27	39	9	0.00	0.7322	3	0.00	0.7322	3
18	23	27	40	9	0.00	0.7322	3	0.00	0.7322	3
18	23	27	41	9	0.00	0.7322	3	0.00	0.7322	3
19	18	23	24	9	0.00	0.7322	3	0.00	0.7322	3
19	18	23	26	9	0.00	0.7322	3	0.00	0.7322	3
19	18	23	27	9	0.00	0.7322	3	0.00	0.7322	3
20	19	18	23	9	0.00	0.6276	3	0.00	0.6276	3
20	19	18	30	9	0.00	-0.2929	3	0.00	-0.2929	3
21	20	19	31	9	0.00	-0.2218	3	0.00	-0.2218	3
21	20	19	32	9	0.00	-0.2218	3	0.00	-0.2218	3
22	20	19	31	9	0.00	-0.2218	3	0.00	-0.2218	3
22	20	19	32	9	0.00	-0.2218	3	0.00	-0.2218	3
23	18	19	31	9	0.00	0.6276	3	0.00	0.6276	3
23	18	19	32	9	0.00	0.6276	3	0.00	0.6276	3
23	24	25	26	9	0.00	0.4184	3	0.00	0.4184	3
23	24	25	35	9	0.00	-0.1213	1	0.00	-0.1213	1
23	24	25	35	9	0.00	0.6402	3	0.00	0.6402	3

23	24	25	36	9	0.00	-0.1213	1	0.00	-0.1213	1
23	24	25	36	9	0.00	0.6402	3	0.00	0.6402	3
23	26	25	24	9	0.00	0.4184	3	0.00	0.4184	3
23	26	25	35	9	0.00	-0.1213	1	0.00	-0.1213	1
23	26	25	35	9	0.00	0.6402	3	0.00	0.6402	3
23	26	25	36	9	0.00	-0.1213	1	0.00	-0.1213	1
23	26	25	36	9	0.00	0.6402	3	0.00	0.6402	3
24	23	18	30	9	0.00	0.7531	3	0.00	0.7531	3
24	23	26	25	9	0.00	0.4184	3	0.00	0.4184	3
24	23	26	37	9	0.00	-0.1213	1	0.00	-0.1213	1
24	23	26	37	9	0.00	0.6402	3	0.00	0.6402	3
24	23	26	38	9	0.00	-0.1213	1	0.00	-0.1213	1
24	23	26	38	9	0.00	0.6402	3	0.00	0.6402	3
24	23	27	39	9	0.00	0.7531	3	0.00	0.7531	3
24	23	27	40	9	0.00	0.7531	3	0.00	0.7531	3
24	23	27	41	9	0.00	0.7531	3	0.00	0.7531	3
24	25	26	37	9	0.00	-0.1213	1	0.00	-0.1213	1
24	25	26	37	9	0.00	0.6402	3	0.00	0.6402	3
24	25	26	38	9	0.00	-0.1213	1	0.00	-0.1213	1
24	25	26	38	9	0.00	0.6402	3	0.00	0.6402	3
25	24	23	26	9	0.00	0.4184	3	0.00	0.4184	3
25	24	23	27	9	0.00	-0.1339	1	0.00	-0.1339	1
25	24	23	27	9	180.00	-0.1339	2	180.00	-0.1339	2
25	24	23	27	9	0.00	0.2929	3	0.00	0.2929	3
25	26	23	27	9	0.00	-0.1339	1	0.00	-0.1339	1
25	26	23	27	9	180.00	-0.1339	2	180.00	-0.1339	2
25	26	23	27	9	0.00	0.2929	3	0.00	0.2929	3
26	23	18	30	9	0.00	0.7531	3	0.00	0.7531	3
26	23	24	33	9	0.00	-0.1213	1	0.00	-0.1213	1
26	23	24	33	9	0.00	0.6402	3	0.00	0.6402	3
26	23	24	34	9	0.00	-0.1213	1	0.00	-0.1213	1
26	23	24	34	9	0.00	0.6402	3	0.00	0.6402	3
26	23	27	39	9	0.00	0.7531	3	0.00	0.7531	3
26	23	27	40	9	0.00	0.7531	3	0.00	0.7531	3
26	23	27	41	9	0.00	0.7531	3	0.00	0.7531	3
26	25	24	33	9	0.00	-0.1213	1	0.00	-0.1213	1
26	25	24	33	9	0.00	0.6402	3	0.00	0.6402	3
26	25	24	34	9	0.00	-0.1213	1	0.00	-0.1213	1
26	25	24	34	9	0.00	0.6402	3	0.00	0.6402	3
27	23	18	30	9	0.00	0.7322	3	0.00	0.7322	3
27	23	24	33	9	0.00	0.1423	1	0.00	0.1423	1
27	23	24	33	9	180.00	0.1715	2	180.00	0.1715	2
27	23	24	33	9	0.00	0.7238	3	0.00	0.7238	3
27	23	24	34	9	0.00	0.1423	1	0.00	0.1423	1
27	23	24	34	9	180.00	0.1715	2	180.00	0.1715	2
27	23	24	34	9	0.00	0.7238	3	0.00	0.7238	3
27	23	26	37	9	0.00	0.1423	1	0.00	0.1423	1
27	23	26	37	9	180.00	0.1715	2	180.00	0.1715	2
27	23	26	37	9	0.00	0.7238	3	0.00	0.7238	3
27	23	26	38	9	0.00	0.1423	1	0.00	0.1423	1
27	23	26	38	9	180.00	0.1715	2	180.00	0.1715	2
27	23	26	38	9	0.00	0.7238	3	0.00	0.7238	3
28	10	9	42	9	180.00	14.6440	2	180.00	14.6440	2
28	10	11	43	9	180.00	14.6440	2	180.00	14.6440	2
30	18	19	31	9	0.00	0.5941	1	0.00	0.5941	1
30	18	19	31	9	180.00	-2.8995	2	180.00	-2.8995	2
30	18	19	31	9	0.00	0.6569	3	0.00	0.6569	3

```
 30  18  19  32 9   0.00   0.5941 1     0.00   0.5941 1
 30  18  19  32 9 180.00  -2.8995 2   180.00  -2.8995 2
 30  18  19  32 9   0.00   0.6569 3     0.00   0.6569 3
 33  24  25  35 9   0.00   0.8870 3     0.00   0.8870 3
 33  24  25  36 9   0.00   0.8870 3     0.00   0.8870 3
 34  24  25  35 9   0.00   0.8870 3     0.00   0.8870 3
 34  24  25  36 9   0.00   0.8870 3     0.00   0.8870 3
 35  25  26  37 9   0.00   0.8870 3     0.00   0.8870 3
 35  25  26  38 9   0.00   0.8870 3     0.00   0.8870 3
 36  25  26  37 9   0.00   0.8870 3     0.00   0.8870 3
 36  25  26  38 9   0.00   0.8870 3     0.00   0.8870 3
 44  14  15  45 9 180.00   8.3680 2   180.00   8.3680 2
```

```
[ dihedrals ]
; ai aj ak al fu xi0 kxi xi0 kxi
  1   2   6  16 2   0.00  21.0790    0.00  21.0790
  2   3   1  29 2   0.00  27.6981    0.00  27.6981
  4   7   3   5 2   0.00  21.0790    0.00  21.0790
  7   8   4  15 2   0.00  -6.6275    0.00  -6.6275
  8   9   7  13 2   0.00  -6.6275    0.00  -6.6275
  9  10   8  42 2   0.00   7.2300    0.00   7.2300
 10  11   9  28 2   0.00  21.0790    0.00  21.0790
 13  14   8  12 2   0.00  30.1081    0.00  30.1081
  6   5   1  17 2   0.00  21.0790    0.00  21.0790
 18  23  17  19 2   0.00   0.0000    0.00   0.0000
 18  19  17  30 2   0.00   0.0000    0.00   0.0000
 19  20  18  32 2   0.00   0.0000    0.00   0.0000
 19  20  18  31 2   0.00   0.0000    0.00   0.0000
 20  21  19  22 2   0.00 107.1941    0.00 107.1941
 23  24  18  26 2   0.00   0.0000    0.00   0.0000
 23  26  18  27 2   0.00   0.0000    0.00   0.0000
 26  25  23  38 2   0.00   0.0000    0.00   0.0000
 26  38  23  37 2   0.00   0.0000    0.00   0.0000
 24  25  23  33 2   0.00   0.0000    0.00   0.0000
 24  33  23  34 2   0.00   0.0000    0.00   0.0000
 25  24  26  35 2   0.00   0.0000    0.00   0.0000
 25  35  26  36 2   0.00   0.0000    0.00   0.0000
 27  40  23  41 2   0.00   0.0000    0.00   0.0000
 27  40  23  39 2   0.00   0.0000    0.00   0.0000
 11  12  10  43 2   0.00  27.6981    0.00  27.6981
 14  15  13  44 2   0.00  -8.4349    0.00  -8.4349
 15  14   7  45 2   0.00  11.4391    0.00  11.4391
```

2) The inhibitor (12):

```
[ atomtypes ]
; name at.num  mass   charge  ptype   sigma         epsilon
NPYD    7  14.0067  0.0  A      0.329632   0.836800
CB      6  12.0110  0.0  A      0.355005   0.292880
C5A     6  12.0110  0.0  A      0.363487   0.209200
NPYL    7  14.0067  0.0  A      0.306469   0.376560
C5B     6  12.0110  0.0  A      0.363487   0.209200
OR      8  15.9994  0.0  A      0.315378   0.636386
F       9  18.9984  0.0  A      0.290433   0.564840
CR      6  12.0110  0.0  A      0.387541   0.230120
NM      7  14.0067  0.0  A      0.329632   0.836800
C=O     6  12.0110  0.0  A      0.356359   0.460240
```

```
O=C     8   15.9994  0.0  A        0.302905    0.502080
NC=O    7   14.0067  0.0  A        0.329632    0.836800
HCMM    1    1.0079  0.0  A        0.235197    0.092048
HOR     1    1.0079  0.0  A        0.040001    0.192464
HNR     1    1.0079  0.0  A        0.040001    0.192464


[ pairtypes ]
; i   j   func   sigma1-4   epsilon1-4 ; THESE ARE 1-4 INTERACTIONS
CR    NPYD  1    0.334087   0.187114
CR    CB    1    0.346773   0.110698
CR    C5A   1    0.351014   0.093557
CR    NPYL  1    0.322505   0.125520
CR    C5B   1    0.351014   0.093557
CR    OR    1    0.326960   0.163176
CR    F     1    0.314487   0.153730
CR    CR    1    0.338541   0.041840
CR    NM    1    0.334087   0.187114
CR    C=O   1    0.347450   0.138768
CR    O=C   1    0.293997   0.144938
CR    NC=O  1    0.334087   0.187114
CR    HCMM  1    0.286869   0.062059
CR    HOR   1    0.189271   0.089737
CR    HNR   1    0.189271   0.089737
O=C   NPYD  1    0.289542   0.648182
O=C   CB    1    0.302228   0.383470
O=C   C5A   1    0.306469   0.324091
O=C   NPYL  1    0.277960   0.434814
O=C   C5B   1    0.306469   0.324091
O=C   OR    1    0.282415   0.565258
O=C   F     1    0.269942   0.532536
O=C   NM    1    0.289542   0.648182
O=C   C=O   1    0.302905   0.480705
O=C   O=C   1    0.249452   0.502080
O=C   NC=O  1    0.289542   0.648182
O=C   HCMM  1    0.242324   0.214978
O=C   HOR   1    0.144726   0.310857
O=C   HNR   1    0.144726   0.310857

[ moleculetype ]
; Name nrexcl
12 3

[ atoms ]
; nr type resnr resid atom cgnr charge mass
   1 NPYD 1  LIG N      1 -0.5670  14.0067
   2 CB   1  LIG C      2 -0.1500  12.0110
   3 CB   1  LIG C1     3  0.1900  12.0110
   4 CB   1  LIG C2     4  0.1600  12.0110
   5 C5A  1  LIG C3     5  0.1054  12.0110
   6 NPYL 1  LIG N1     6  0.0332  14.0067
   7 C5A  1  LIG CA     7 -0.3316  12.0110
   8 C5B  1  LIG C5     8 -0.0540  12.0110
   9 C5B  1  LIG C6     9  0.0000  12.0110
  10 CB   1  LIG C7    10  0.6740  12.0110
  11 NPYD 1  LIG N2    11 -0.6200  14.0067
  12 CB   1  LIG C8    12  0.0580  12.0110
```

```
  13 CB   1  LIG C9     13  0.1900  12.0110
  14 CB   1  LIG C10    14  0.1600  12.0110
  15 NPYD 1  LIG N3     15 -0.6200  14.0067
  16 OR   1  LIG O      16 -0.6800  15.9994
  17 F    1  LIG F      17 -0.1900  18.9984
  18 CR   1  LIG C4     18  0.4600  12.0110
  19 F    1  LIG F1     19 -0.1900  18.9984
  20 NM   1  LIG N4     20 -0.6980  14.0067
  21 CR   1  LIG C11    21 -0.0500  12.0110
  22 CR   1  LIG C12    22  0.0000  12.0110
  23 CR   1  LIG C13    23 -0.0500  12.0110
  24 CR   1  LIG C14    24  0.0000  12.0110
  25 CR   1  LIG C15    25  0.0000  12.0110
  26 CR   1  LIG C16    26  0.0000  12.0110
  27 NM   1  LIG N5     27 -0.7300  14.0067
  28 C=O  1  LIG C17    28  0.4100  12.0110
  29 O=C  1  LIG O1     29 -0.5700  15.9994
  30 NC=O 1  LIG N6     30 -0.6602  14.0067
  31 CR   1  LIG C18    31  0.3001  12.0110
  32 CR   1  LIG C19    32  0.0000  12.0110
  33 CR   1  LIG C20    33  0.0000  12.0110
  34 CR   1  LIG C21    34  0.3001  12.0110
  35 HCMM 1  LIG H      35  0.1500   1.0079
  36 HOR  1  LIG H3     36  0.4000   1.0079
  37 HCMM 1  LIG H4     37  0.1500   1.0079
  38 HNR  1  LIG H5     38  0.2700   1.0079
  39 HCMM 1  LIG H1     39  0.0000   1.0079
  40 HCMM 1  LIG H2     40  0.0000   1.0079
  41 HCMM 1  LIG H6     41  0.1500   1.0079
  42 HCMM 1  LIG H7     42  0.0000   1.0079
  43 HCMM 1  LIG H8     43  0.0000   1.0079
  44 HCMM 1  LIG H9     44  0.0000   1.0079
  45 HCMM 1  LIG H10    45  0.0000   1.0079
  46 HCMM 1  LIG H11    46  0.0000   1.0079
  47 HCMM 1  LIG H12    47  0.0000   1.0079
  48 HCMM 1  LIG H13    48  0.0000   1.0079
  49 HCMM 1  LIG H14    49  0.0000   1.0079
  50 HCMM 1  LIG H15    50  0.0000   1.0079
  51 HCMM 1  LIG H16    51  0.0000   1.0079
  52 HCMM 1  LIG H17    52  0.0000   1.0079
  53 HCMM 1  LIG H18    53  0.0000   1.0079
  54 HCMM 1  LIG H19    54  0.0000   1.0079
  55 HCMM 1  LIG H20    55  0.0000   1.0079
  56 HCMM 1  LIG H21    56  0.0000   1.0079
  57 HCMM 1  LIG H22    57  0.0000   1.0079
  58 HCMM 1  LIG H23    58  0.0000   1.0079
  59 HCMM 1  LIG H24    59 -0.0000   1.0079

[ bonds ]
; ai aj fu b0 kb, b0 kb
 38   6 1 0.10120  428294.3  0.10120  428294.3
 36  16 1 0.09720  469365.3  0.09720  469365.3
 16  18 1 0.14180  303937.5  0.14180  303937.5
  6   5 1 0.13640  379454.5  0.13640  379454.5
  6   7 1 0.13640  379454.5  0.13640  379454.5
  1   5 1 0.13300  439556.0  0.13300  439556.0
  1   4 1 0.13330  345489.6  0.13330  345489.6
```

39	18	1	0.10930	287014.9	0.10930	287014.9
37	4	1	0.10840	319534.6	0.10840	319534.6
18	7	1	0.14710	269852.1	0.14710	269852.1
18	40	1	0.10930	287014.9	0.10930	287014.9
5	9	1	0.13770	428655.8	0.13770	428655.8
4	3	1	0.13740	335613.7	0.13740	335613.7
7	8	1	0.13770	428655.8	0.13770	428655.8
9	8	1	0.14180	259734.4	0.14180	259734.4
9	2	1	0.13790	371023.7	0.13790	371023.7
8	10	1	0.13790	371023.7	0.13790	371023.7
3	2	1	0.13740	335613.7	0.13740	335613.7
3	17	1	0.13420	392101.0	0.13420	392101.0
2	35	1	0.10840	319534.6	0.10840	319534.6
15	10	1	0.13330	345489.6	0.13330	345489.6
15	14	1	0.13330	345489.6	0.13330	345489.6
10	11	1	0.13330	345489.6	0.13330	345489.6
11	12	1	0.13330	345489.6	0.13330	345489.6
14	41	1	0.10840	319534.6	0.10840	319534.6
14	13	1	0.13740	335613.7	0.13740	335613.7
12	13	1	0.13740	335613.7	0.13740	335613.7
12	20	1	0.13350	429799.7	0.13350	429799.7
42	21	1	0.10930	287014.9	0.10930	287014.9
13	19	1	0.13420	392101.0	0.13420	392101.0
48	25	1	0.10930	287014.9	0.10930	287014.9
51	26	1	0.10930	287014.9	0.10930	287014.9
21	20	1	0.14440	268346.7	0.14440	268346.7
21	26	1	0.15080	256422.3	0.15080	256422.3
21	22	1	0.15080	256422.3	0.15080	256422.3
45	23	1	0.10930	287014.9	0.10930	287014.9
29	28	1	0.12220	779866.6	0.12220	779866.6
26	25	1	0.15080	256422.3	0.15080	256422.3
26	50	1	0.10930	287014.9	0.10930	287014.9
43	22	1	0.10930	287014.9	0.10930	287014.9
25	49	1	0.10930	287014.9	0.10930	287014.9
25	24	1	0.15080	256422.3	0.15080	256422.3
22	23	1	0.15080	256422.3	0.15080	256422.3
22	44	1	0.10930	287014.9	0.10930	287014.9
23	24	1	0.15080	256422.3	0.15080	256422.3
23	27	1	0.14440	268346.7	0.14440	268346.7
53	31	1	0.10930	287014.9	0.10930	287014.9
46	24	1	0.10930	287014.9	0.10930	287014.9
24	47	1	0.10930	287014.9	0.10930	287014.9
28	27	1	0.13220	455755.6	0.13220	455755.6
28	30	1	0.13690	351030.1	0.13690	351030.1
31	52	1	0.10930	287014.9	0.10930	287014.9
31	30	1	0.14360	280872.8	0.14360	280872.8
31	32	1	0.15080	256422.3	0.15080	256422.3
30	34	1	0.14360	280872.8	0.14360	280872.8
55	32	1	0.10930	287014.9	0.10930	287014.9
32	54	1	0.10930	287014.9	0.10930	287014.9
32	33	1	0.15080	256422.3	0.15080	256422.3
58	34	1	0.10930	287014.9	0.10930	287014.9
34	59	1	0.10930	287014.9	0.10930	287014.9
34	33	1	0.15080	256422.3	0.15080	256422.3
33	56	1	0.10930	287014.9	0.10930	287014.9
33	57	1	0.10930	287014.9	0.10930	287014.9

```
[ pairs ]
; ai aj fu
  1   2 1
  1  17 1
  1   7 1
  1  38 1
  1   8 1
  2  37 1
  2   6 1
  2   7 1
  2  10 1
  3   5 1
  3   8 1
  4   6 1
  4   9 1
  4  35 1
  5  37 1
  5  18 1
  5  35 1
  5  10 1
  6  10 1
  6  16 1
  6  39 1
  6  40 1
  7  11 1
  7  15 1
  7  36 1
  8  38 1
  8  16 1
  8  39 1
  8  40 1
  8  35 1
  8  12 1
  8  14 1
  9  17 1
  9  38 1
  9  18 1
  9  11 1
  9  15 1
 10  18 1
 10  13 1
 10  20 1
 10  41 1
 11  14 1
 11  19 1
 11  21 1
 12  15 1
 12  41 1
 12  22 1
 12  26 1
 12  42 1
 13  21 1
 14  20 1
 15  19 1
 17  35 1
 17  37 1
 18  38 1
```

19 20 1
19 41 1
20 23 1
20 43 1
20 44 1
20 25 1
20 50 1
20 51 1
21 24 1
21 27 1
21 45 1
21 48 1
21 49 1
22 25 1
22 50 1
22 51 1
22 46 1
22 47 1
22 28 1
23 26 1
23 42 1
23 48 1
23 49 1
23 29 1
23 30 1
24 43 1
24 44 1
24 28 1
24 50 1
24 51 1
25 27 1
25 45 1
25 42 1
26 43 1
26 44 1
26 46 1
26 47 1
27 43 1
27 44 1
27 46 1
27 47 1
27 31 1
27 34 1
28 45 1
28 32 1
28 52 1
28 53 1
28 33 1
28 58 1
28 59 1
29 31 1
29 34 1
30 54 1
30 55 1
30 56 1
30 57 1
31 58 1

```
 31  59 1
 31  56 1
 31  57 1
 32  58 1
 32  59 1
 33  52 1
 33  53 1
 34  52 1
 34  53 1
 34  54 1
 34  55 1
 36  39 1
 36  40 1
 42  43 1
 42  44 1
 42  50 1
 42  51 1
 43  45 1
 44  45 1
 45  46 1
 45  47 1
 46  48 1
 46  49 1
 47  48 1
 47  49 1
 48  50 1
 48  51 1
 49  50 1
 49  51 1
 52  54 1
 52  55 1
 53  54 1
 53  55 1
 54  56 1
 54  57 1
 55  56 1
 55  57 1
 56  58 1
 56  59 1
 57  58 1
 57  59 1

[ angles ]
; ai aj ak fu th0 kth ub0 kub th0 kth ub0 kub
  4   1   5 1  110.1810  740.72    110.1810  740.72
  3   2   9 1  112.5670  254.73    112.5670  254.73
  3   2  35 1  120.5710  339.05    120.5710  339.05
  9   2  35 1  121.4460  314.95    121.4460  314.95
  2   3   4 1  119.9770  402.88    119.9770  402.88
  2   3  17 1  118.0650  658.81    118.0650  658.81
  4   3  17 1  118.0650  658.81    118.0650  658.81
  1   4   3 1  126.1390  358.92    126.1390  358.92
  1   4  37 1  115.5880  417.33    115.5880  417.33
  3   4  37 1  120.5710  339.05    120.5710  339.05
  1   5   6 1  124.8140  615.46    124.8140  615.46
  1   5   9 1  126.5130  548.01    126.5130  548.01
  6   5   9 1  107.2550  489.59    107.2550  489.59
```

5	6	7	1	109.5990	693.74	109.5990	693.74
5	6	38	1	127.7700	331.82	127.7700	331.82
7	6	38	1	127.7700	331.82	127.7700	331.82
6	7	8	1	107.2550	489.59	107.2550	489.59
6	7	18	1	121.8320	563.07	121.8320	563.07
8	7	18	1	131.3780	443.83	131.3780	443.83
7	8	9	1	108.2390	521.51	108.2390	521.51
7	8	10	1	120.0000	602.21	120.0000	602.21
9	8	10	1	128.6730	464.91	128.6730	464.91
2	9	5	1	120.0000	602.21	120.0000	602.21
2	9	8	1	128.6730	464.91	128.6730	464.91
5	9	8	1	108.2390	521.51	108.2390	521.51
8	10	11	1	128.6730	464.91	128.6730	464.91
8	10	15	1	128.6730	464.91	128.6730	464.91
11	10	15	1	128.9380	436.60	128.9380	436.60
10	11	12	1	115.4060	653.40	115.4060	653.40
11	12	13	1	126.1390	358.92	126.1390	358.92
11	12	20	1	118.3490	691.34	118.3490	691.34
13	12	20	1	124.3840	566.68	124.3840	566.68
12	13	14	1	119.9770	402.88	119.9770	402.88
12	13	19	1	118.0650	658.81	118.0650	658.81
14	13	19	1	118.0650	658.81	118.0650	658.81
13	14	15	1	126.1390	358.92	126.1390	358.92
13	14	41	1	120.5710	339.05	120.5710	339.05
15	14	41	1	115.5880	417.33	115.5880	417.33
10	15	14	1	115.4060	653.40	115.4060	653.40
18	16	36	1	106.5030	477.55	106.5030	477.55
7	18	16	1	106.5350	813.59	106.5350	813.59
7	18	39	1	110.4670	373.97	110.4670	373.97
7	18	40	1	110.4670	373.97	110.4670	373.97
16	18	39	1	108.5770	470.32	108.5770	470.32
16	18	40	1	108.5770	470.32	108.5770	470.32
39	18	40	1	108.8360	310.74	108.8360	310.74
12	20	21	1	120.0000	602.21	120.0000	602.21
20	21	22	1	109.5000	602.21	109.5000	602.21
20	21	26	1	109.5000	602.21	109.5000	602.21
20	21	42	1	113.0350	394.45	113.0350	394.45
22	21	26	1	109.6080	512.48	109.6080	512.48
22	21	42	1	110.5490	383.00	110.5490	383.00
26	21	42	1	110.5490	383.00	110.5490	383.00
21	22	23	1	109.6080	512.48	109.6080	512.48
21	22	43	1	110.5490	383.00	110.5490	383.00
21	22	44	1	110.5490	383.00	110.5490	383.00
23	22	43	1	110.5490	383.00	110.5490	383.00
23	22	44	1	110.5490	383.00	110.5490	383.00
43	22	44	1	108.8360	310.74	108.8360	310.74
22	23	24	1	109.6080	512.48	109.6080	512.48
22	23	27	1	109.5000	602.21	109.5000	602.21
22	23	45	1	110.5490	383.00	110.5490	383.00
24	23	27	1	109.5000	602.21	109.5000	602.21
24	23	45	1	110.5490	383.00	110.5490	383.00
27	23	45	1	113.0350	394.45	113.0350	394.45
23	24	25	1	109.6080	512.48	109.6080	512.48
23	24	46	1	110.5490	383.00	110.5490	383.00
23	24	47	1	110.5490	383.00	110.5490	383.00
25	24	46	1	110.5490	383.00	110.5490	383.00
25	24	47	1	110.5490	383.00	110.5490	383.00

46 24 47 1 108.8360 310.74 108.8360 310.74
24 25 26 1 109.6080 512.48 109.6080 512.48
24 25 48 1 110.5490 383.00 110.5490 383.00
24 25 49 1 110.5490 383.00 110.5490 383.00
26 25 48 1 110.5490 383.00 110.5490 383.00
26 25 49 1 110.5490 383.00 110.5490 383.00
48 25 49 1 108.8360 310.74 108.8360 310.74
21 26 25 1 109.6080 512.48 109.6080 512.48
21 26 50 1 110.5490 383.00 110.5490 383.00
21 26 51 1 110.5490 383.00 110.5490 383.00
25 26 50 1 110.5490 383.00 110.5490 383.00
25 26 51 1 110.5490 383.00 110.5490 383.00
50 26 51 1 108.8360 310.74 108.8360 310.74
23 27 28 1 120.0000 602.21 120.0000 602.21
27 28 29 1 129.3490 679.89 129.3490 679.89
27 28 30 1 120.0000 602.21 120.0000 602.21
29 28 30 1 127.1520 546.20 127.1520 546.20
28 30 31 1 119.6000 494.41 119.6000 494.41
28 30 34 1 119.6000 494.41 119.6000 494.41
31 30 34 1 117.9090 672.67 117.9090 672.67
30 31 32 1 109.9600 632.32 109.9600 632.32
30 31 52 1 107.6460 445.64 107.6460 445.64
30 31 53 1 107.6460 445.64 107.6460 445.64
32 31 52 1 110.5490 383.00 110.5490 383.00
32 31 53 1 110.5490 383.00 110.5490 383.00
52 31 53 1 108.8360 310.74 108.8360 310.74
31 32 33 1 109.6080 512.48 109.6080 512.48
31 32 54 1 110.5490 383.00 110.5490 383.00
31 32 55 1 110.5490 383.00 110.5490 383.00
33 32 54 1 110.5490 383.00 110.5490 383.00
33 32 55 1 110.5490 383.00 110.5490 383.00
54 32 55 1 108.8360 310.74 108.8360 310.74
32 33 34 1 109.6080 512.48 109.6080 512.48
32 33 56 1 110.5490 383.00 110.5490 383.00
32 33 57 1 110.5490 383.00 110.5490 383.00
34 33 56 1 110.5490 383.00 110.5490 383.00
34 33 57 1 110.5490 383.00 110.5490 383.00
56 33 57 1 108.8360 310.74 108.8360 310.74
30 34 33 1 109.9600 632.32 109.9600 632.32
30 34 58 1 107.6460 445.64 107.6460 445.64
30 34 59 1 107.6460 445.64 107.6460 445.64
33 34 58 1 110.5490 383.00 110.5490 383.00
33 34 59 1 110.5490 383.00 110.5490 383.00
58 34 59 1 108.8360 310.74 108.8360 310.74

[dihedrals]
; ai aj ak al fu phi0 kphi mult phi0 kphi mult
1 4 3 2 9 180.00 14.6440 2 180.00 14.6440 2
1 4 3 17 9 180.00 14.6440 2 180.00 14.6440 2
1 5 6 7 9 180.00 8.3680 2 180.00 8.3680 2
1 5 6 38 9 180.00 8.3680 2 180.00 8.3680 2
1 5 9 2 9 180.00 14.6440 2 180.00 14.6440 2
1 5 9 8 9 180.00 14.6440 2 180.00 14.6440 2
2 3 4 37 9 180.00 14.6440 2 180.00 14.6440 2
2 9 5 6 9 180.00 14.6440 2 180.00 14.6440 2
2 9 8 7 9 180.00 14.6440 2 180.00 14.6440 2
2 9 8 10 9 180.00 14.6440 2 180.00 14.6440 2

3	2	9	5	9	180.00	14.6440	2	180.00	14.6440	2
3	2	9	8	9	180.00	14.6440	2	180.00	14.6440	2
3	4	1	5	9	180.00	14.6440	2	180.00	14.6440	2
4	1	5	6	9	180.00	14.6440	2	180.00	14.6440	2
4	1	5	9	9	180.00	14.6440	2	180.00	14.6440	2
4	3	2	9	9	180.00	14.6440	2	180.00	14.6440	2
4	3	2	35	9	180.00	14.6440	2	180.00	14.6440	2
5	1	4	37	9	180.00	14.6440	2	180.00	14.6440	2
5	6	7	8	9	180.00	8.3680	2	180.00	8.3680	2
5	6	7	18	9	180.00	8.3680	2	180.00	8.3680	2
5	9	2	35	9	180.00	14.6440	2	180.00	14.6440	2
5	9	8	7	9	180.00	14.6440	2	180.00	14.6440	2
5	9	8	10	9	180.00	14.6440	2	180.00	14.6440	2
6	5	9	8	9	180.00	14.6440	2	180.00	14.6440	2
6	7	8	9	9	180.00	14.6440	2	180.00	14.6440	2
6	7	8	10	9	180.00	14.6440	2	180.00	14.6440	2
7	6	5	9	9	180.00	8.3680	2	180.00	8.3680	2
7	8	10	11	9	180.00	3.7656	2	180.00	3.7656	2
7	8	10	15	9	180.00	3.7656	2	180.00	3.7656	2
7	18	16	36	9	0.00	0.4184	3	0.00	0.4184	3
8	7	6	38	9	180.00	8.3680	2	180.00	8.3680	2
8	9	2	35	9	180.00	14.6440	2	180.00	14.6440	2
9	2	3	17	9	180.00	14.6440	2	180.00	14.6440	2
9	5	6	38	9	180.00	8.3680	2	180.00	8.3680	2
9	8	7	18	9	180.00	14.6440	2	180.00	14.6440	2
9	8	10	11	9	180.00	3.7656	2	180.00	3.7656	2
9	8	10	15	9	180.00	3.7656	2	180.00	3.7656	2
10	11	12	13	9	180.00	14.6440	2	180.00	14.6440	2
10	11	12	20	9	180.00	14.6440	2	180.00	14.6440	2
10	15	14	13	9	180.00	14.6440	2	180.00	14.6440	2
10	15	14	41	9	180.00	14.6440	2	180.00	14.6440	2
11	10	15	14	9	180.00	14.6440	2	180.00	14.6440	2
11	12	13	14	9	180.00	14.6440	2	180.00	14.6440	2
11	12	13	19	9	180.00	14.6440	2	180.00	14.6440	2
11	12	20	21	9	180.00	7.5312	2	180.00	7.5312	2
12	11	10	15	9	180.00	14.6440	2	180.00	14.6440	2
12	13	14	15	9	180.00	14.6440	2	180.00	14.6440	2
12	13	14	41	9	180.00	14.6440	2	180.00	14.6440	2
12	20	21	22	9	0.00	0.5230	3	0.00	0.5230	3
12	20	21	26	9	0.00	0.5230	3	0.00	0.5230	3
12	20	21	42	9	0.00	0.5230	3	0.00	0.5230	3
13	12	20	21	9	180.00	7.5312	2	180.00	7.5312	2
14	13	12	20	9	180.00	14.6440	2	180.00	14.6440	2
15	14	13	19	9	180.00	14.6440	2	180.00	14.6440	2
17	3	2	35	9	180.00	14.6440	2	180.00	14.6440	2
17	3	4	37	9	180.00	14.6440	2	180.00	14.6440	2
18	7	6	38	9	180.00	8.3680	2	180.00	8.3680	2
19	13	12	20	9	180.00	14.6440	2	180.00	14.6440	2
19	13	14	41	9	180.00	14.6440	2	180.00	14.6440	2
20	21	22	23	9	0.00	0.6276	3	0.00	0.6276	3
20	21	22	43	9	0.00	0.6276	3	0.00	0.6276	3
20	21	22	44	9	0.00	0.6276	3	0.00	0.6276	3
20	21	26	25	9	0.00	0.6276	3	0.00	0.6276	3
20	21	26	50	9	0.00	0.6276	3	0.00	0.6276	3
20	21	26	51	9	0.00	0.6276	3	0.00	0.6276	3
21	22	23	24	9	0.00	0.2134	1	0.00	0.2134	1
21	22	23	24	9	180.00	1.4267	2	180.00	1.4267	2

21	22	23	24	9	0.00	0.6945	3	0.00	0.6945	3
21	22	23	27	9	0.00	0.6276	3	0.00	0.6276	3
21	22	23	45	9	0.00	1.3389	1	0.00	1.3389	1
21	22	23	45	9	180.00	-1.3180	2	180.00	-1.3180	2
21	22	23	45	9	0.00	0.5523	3	0.00	0.5523	3
21	26	25	24	9	0.00	0.2134	1	0.00	0.2134	1
21	26	25	24	9	180.00	1.4267	2	180.00	1.4267	2
21	26	25	24	9	0.00	0.6945	3	0.00	0.6945	3
21	26	25	48	9	0.00	1.3389	1	0.00	1.3389	1
21	26	25	48	9	180.00	-1.3180	2	180.00	-1.3180	2
21	26	25	48	9	0.00	0.5523	3	0.00	0.5523	3
21	26	25	49	9	0.00	1.3389	1	0.00	1.3389	1
21	26	25	49	9	180.00	-1.3180	2	180.00	-1.3180	2
21	26	25	49	9	0.00	0.5523	3	0.00	0.5523	3
22	21	26	25	9	0.00	0.2134	1	0.00	0.2134	1
22	21	26	25	9	180.00	1.4267	2	180.00	1.4267	2
22	21	26	25	9	0.00	0.6945	3	0.00	0.6945	3
22	21	26	50	9	0.00	1.3389	1	0.00	1.3389	1
22	21	26	50	9	180.00	-1.3180	2	180.00	-1.3180	2
22	21	26	50	9	0.00	0.5523	3	0.00	0.5523	3
22	21	26	51	9	0.00	1.3389	1	0.00	1.3389	1
22	21	26	51	9	180.00	-1.3180	2	180.00	-1.3180	2
22	21	26	51	9	0.00	0.5523	3	0.00	0.5523	3
22	23	24	25	9	0.00	0.2134	1	0.00	0.2134	1
22	23	24	25	9	180.00	1.4267	2	180.00	1.4267	2
22	23	24	25	9	0.00	0.6945	3	0.00	0.6945	3
22	23	24	46	9	0.00	1.3389	1	0.00	1.3389	1
22	23	24	46	9	180.00	-1.3180	2	180.00	-1.3180	2
22	23	24	46	9	0.00	0.5523	3	0.00	0.5523	3
22	23	24	47	9	0.00	1.3389	1	0.00	1.3389	1
22	23	24	47	9	180.00	-1.3180	2	180.00	-1.3180	2
22	23	24	47	9	0.00	0.5523	3	0.00	0.5523	3
22	23	27	28	9	0.00	0.5230	3	0.00	0.5230	3
23	22	21	26	9	0.00	0.2134	1	0.00	0.2134	1
23	22	21	26	9	180.00	1.4267	2	180.00	1.4267	2
23	22	21	26	9	0.00	0.6945	3	0.00	0.6945	3
23	22	21	42	9	0.00	1.3389	1	0.00	1.3389	1
23	22	21	42	9	180.00	-1.3180	2	180.00	-1.3180	2
23	22	21	42	9	0.00	0.5523	3	0.00	0.5523	3
23	24	25	26	9	0.00	0.2134	1	0.00	0.2134	1
23	24	25	26	9	180.00	1.4267	2	180.00	1.4267	2
23	24	25	26	9	0.00	0.6945	3	0.00	0.6945	3
23	24	25	48	9	0.00	1.3389	1	0.00	1.3389	1
23	24	25	48	9	180.00	-1.3180	2	180.00	-1.3180	2
23	24	25	48	9	0.00	0.5523	3	0.00	0.5523	3
23	24	25	49	9	0.00	1.3389	1	0.00	1.3389	1
23	24	25	49	9	180.00	-1.3180	2	180.00	-1.3180	2
23	24	25	49	9	0.00	0.5523	3	0.00	0.5523	3
23	27	28	29	9	180.00	7.5312	2	180.00	7.5312	2
23	27	28	30	9	180.00	7.5312	2	180.00	7.5312	2
24	23	22	43	9	0.00	1.3389	1	0.00	1.3389	1
24	23	22	43	9	180.00	-1.3180	2	180.00	-1.3180	2
24	23	22	43	9	0.00	0.5523	3	0.00	0.5523	3
24	23	22	44	9	0.00	1.3389	1	0.00	1.3389	1
24	23	22	44	9	180.00	-1.3180	2	180.00	-1.3180	2
24	23	22	44	9	0.00	0.5523	3	0.00	0.5523	3
24	23	27	28	9	0.00	0.5230	3	0.00	0.5230	3

24	25	26	50	9	0.00	1.3389	1	0.00	1.3389	1
24	25	26	50	9	180.00	-1.3180	2	180.00	-1.3180	2
24	25	26	50	9	0.00	0.5523	3	0.00	0.5523	3
24	25	26	51	9	0.00	1.3389	1	0.00	1.3389	1
24	25	26	51	9	180.00	-1.3180	2	180.00	-1.3180	2
24	25	26	51	9	0.00	0.5523	3	0.00	0.5523	3
25	24	23	27	9	0.00	0.6276	3	0.00	0.6276	3
25	24	23	45	9	0.00	1.3389	1	0.00	1.3389	1
25	24	23	45	9	180.00	-1.3180	2	180.00	-1.3180	2
25	24	23	45	9	0.00	0.5523	3	0.00	0.5523	3
25	26	21	42	9	0.00	1.3389	1	0.00	1.3389	1
25	26	21	42	9	180.00	-1.3180	2	180.00	-1.3180	2
25	26	21	42	9	0.00	0.5523	3	0.00	0.5523	3
26	21	22	43	9	0.00	1.3389	1	0.00	1.3389	1
26	21	22	43	9	180.00	-1.3180	2	180.00	-1.3180	2
26	21	22	43	9	0.00	0.5523	3	0.00	0.5523	3
26	21	22	44	9	0.00	1.3389	1	0.00	1.3389	1
26	21	22	44	9	180.00	-1.3180	2	180.00	-1.3180	2
26	21	22	44	9	0.00	0.5523	3	0.00	0.5523	3
26	25	24	46	9	0.00	1.3389	1	0.00	1.3389	1
26	25	24	46	9	180.00	-1.3180	2	180.00	-1.3180	2
26	25	24	46	9	0.00	0.5523	3	0.00	0.5523	3
26	25	24	47	9	0.00	1.3389	1	0.00	1.3389	1
26	25	24	47	9	180.00	-1.3180	2	180.00	-1.3180	2
26	25	24	47	9	0.00	0.5523	3	0.00	0.5523	3
27	23	22	43	9	0.00	0.6276	3	0.00	0.6276	3
27	23	22	44	9	0.00	0.6276	3	0.00	0.6276	3
27	23	24	46	9	0.00	0.6276	3	0.00	0.6276	3
27	23	24	47	9	0.00	0.6276	3	0.00	0.6276	3
27	28	30	31	9	180.00	12.5520	2	180.00	12.5520	2
27	28	30	34	9	180.00	12.5520	2	180.00	12.5520	2
28	27	23	45	9	0.00	0.5230	3	0.00	0.5230	3
28	30	31	32	9	0.00	-2.1464	1	0.00	-2.1464	1
28	30	31	32	9	180.00	1.4518	2	180.00	1.4518	2
28	30	31	32	9	0.00	1.9832	3	0.00	1.9832	3
28	30	31	52	9	0.00	-4.3932	1	0.00	-4.3932	1
28	30	31	52	9	180.00	2.8493	2	180.00	2.8493	2
28	30	31	52	9	0.00	0.0460	3	0.00	0.0460	3
28	30	31	53	9	0.00	-4.3932	1	0.00	-4.3932	1
28	30	31	53	9	180.00	2.8493	2	180.00	2.8493	2
28	30	31	53	9	0.00	0.0460	3	0.00	0.0460	3
28	30	34	33	9	0.00	-2.1464	1	0.00	-2.1464	1
28	30	34	33	9	180.00	1.4518	2	180.00	1.4518	2
28	30	34	33	9	0.00	1.9832	3	0.00	1.9832	3
28	30	34	58	9	0.00	-4.3932	1	0.00	-4.3932	1
28	30	34	58	9	180.00	2.8493	2	180.00	2.8493	2
28	30	34	58	9	0.00	0.0460	3	0.00	0.0460	3
28	30	34	59	9	0.00	-4.3932	1	0.00	-4.3932	1
28	30	34	59	9	180.00	2.8493	2	180.00	2.8493	2
28	30	34	59	9	0.00	0.0460	3	0.00	0.0460	3
29	28	30	31	9	0.00	-0.6694	1	0.00	-0.6694	1
29	28	30	31	9	180.00	13.1670	2	180.00	13.1670	2
29	28	30	31	9	0.00	-0.3054	3	0.00	-0.3054	3
29	28	30	34	9	0.00	-0.6694	1	0.00	-0.6694	1
29	28	30	34	9	180.00	13.1670	2	180.00	13.1670	2
29	28	30	34	9	0.00	-0.3054	3	0.00	-0.3054	3
30	31	32	33	9	0.00	0.6276	3	0.00	0.6276	3

30	31	32	54	9	0.00	0.8912	3	0.00	0.8912	3
30	31	32	55	9	0.00	0.8912	3	0.00	0.8912	3
30	34	33	32	9	0.00	0.6276	3	0.00	0.6276	3
30	34	33	56	9	0.00	0.8912	3	0.00	0.8912	3
30	34	33	57	9	0.00	0.8912	3	0.00	0.8912	3
31	30	34	33	9	0.00	0.6276	3	0.00	0.6276	3
31	30	34	58	9	0.00	1.6318	3	0.00	1.6318	3
31	30	34	59	9	0.00	1.6318	3	0.00	1.6318	3
31	32	33	34	9	0.00	0.2134	1	0.00	0.2134	1
31	32	33	34	9	180.00	1.4267	2	180.00	1.4267	2
31	32	33	34	9	0.00	0.6945	3	0.00	0.6945	3
31	32	33	56	9	0.00	1.3389	1	0.00	1.3389	1
31	32	33	56	9	180.00	-1.3180	2	180.00	-1.3180	2
31	32	33	56	9	0.00	0.5523	3	0.00	0.5523	3
31	32	33	57	9	0.00	1.3389	1	0.00	1.3389	1
31	32	33	57	9	180.00	-1.3180	2	180.00	-1.3180	2
31	32	33	57	9	0.00	0.5523	3	0.00	0.5523	3
32	31	30	34	9	0.00	0.6276	3	0.00	0.6276	3
32	33	34	58	9	0.00	1.3389	1	0.00	1.3389	1
32	33	34	58	9	180.00	-1.3180	2	180.00	-1.3180	2
32	33	34	58	9	0.00	0.5523	3	0.00	0.5523	3
32	33	34	59	9	0.00	1.3389	1	0.00	1.3389	1
32	33	34	59	9	180.00	-1.3180	2	180.00	-1.3180	2
32	33	34	59	9	0.00	0.5523	3	0.00	0.5523	3
33	32	31	52	9	0.00	1.3389	1	0.00	1.3389	1
33	32	31	52	9	180.00	-1.3180	2	180.00	-1.3180	2
33	32	31	52	9	0.00	0.5523	3	0.00	0.5523	3
33	32	31	53	9	0.00	1.3389	1	0.00	1.3389	1
33	32	31	53	9	180.00	-1.3180	2	180.00	-1.3180	2
33	32	31	53	9	0.00	0.5523	3	0.00	0.5523	3
34	30	31	52	9	0.00	1.6318	3	0.00	1.6318	3
34	30	31	53	9	0.00	1.6318	3	0.00	1.6318	3
34	33	32	54	9	0.00	1.3389	1	0.00	1.3389	1
34	33	32	54	9	180.00	-1.3180	2	180.00	-1.3180	2
34	33	32	54	9	0.00	0.5523	3	0.00	0.5523	3
34	33	32	55	9	0.00	1.3389	1	0.00	1.3389	1
34	33	32	55	9	180.00	-1.3180	2	180.00	-1.3180	2
34	33	32	55	9	0.00	0.5523	3	0.00	0.5523	3
36	16	18	39	9	0.00	1.2468	1	0.00	1.2468	1
36	16	18	39	9	180.00	-0.5774	2	180.00	-0.5774	2
36	16	18	39	9	0.00	0.7238	3	0.00	0.7238	3
36	16	18	40	9	0.00	1.2468	1	0.00	1.2468	1
36	16	18	40	9	180.00	-0.5774	2	180.00	-0.5774	2
36	16	18	40	9	0.00	0.7238	3	0.00	0.7238	3
42	21	22	43	9	0.00	0.5941	1	0.00	0.5941	1
42	21	22	43	9	180.00	-2.8995	2	180.00	-2.8995	2
42	21	22	43	9	0.00	0.6569	3	0.00	0.6569	3
42	21	22	44	9	0.00	0.5941	1	0.00	0.5941	1
42	21	22	44	9	180.00	-2.8995	2	180.00	-2.8995	2
42	21	22	44	9	0.00	0.6569	3	0.00	0.6569	3
42	21	26	50	9	0.00	0.5941	1	0.00	0.5941	1
42	21	26	50	9	180.00	-2.8995	2	180.00	-2.8995	2
42	21	26	50	9	0.00	0.6569	3	0.00	0.6569	3
42	21	26	51	9	0.00	0.5941	1	0.00	0.5941	1
42	21	26	51	9	180.00	-2.8995	2	180.00	-2.8995	2
42	21	26	51	9	0.00	0.6569	3	0.00	0.6569	3
43	22	23	45	9	0.00	0.5941	1	0.00	0.5941	1

43	22	23	45	9	180.00	-2.8995	2	180.00	-2.8995	2
43	22	23	45	9	0.00	0.6569	3	0.00	0.6569	3
44	22	23	45	9	0.00	0.5941	1	0.00	0.5941	1
44	22	23	45	9	180.00	-2.8995	2	180.00	-2.8995	2
44	22	23	45	9	0.00	0.6569	3	0.00	0.6569	3
45	23	24	46	9	0.00	0.5941	1	0.00	0.5941	1
45	23	24	46	9	180.00	-2.8995	2	180.00	-2.8995	2
45	23	24	46	9	0.00	0.6569	3	0.00	0.6569	3
45	23	24	47	9	0.00	0.5941	1	0.00	0.5941	1
45	23	24	47	9	180.00	-2.8995	2	180.00	-2.8995	2
45	23	24	47	9	0.00	0.6569	3	0.00	0.6569	3
46	24	25	48	9	0.00	0.5941	1	0.00	0.5941	1
46	24	25	48	9	180.00	-2.8995	2	180.00	-2.8995	2
46	24	25	48	9	0.00	0.6569	3	0.00	0.6569	3
46	24	25	49	9	0.00	0.5941	1	0.00	0.5941	1
46	24	25	49	9	180.00	-2.8995	2	180.00	-2.8995	2
46	24	25	49	9	0.00	0.6569	3	0.00	0.6569	3
47	24	25	48	9	0.00	0.5941	1	0.00	0.5941	1
47	24	25	48	9	180.00	-2.8995	2	180.00	-2.8995	2
47	24	25	48	9	0.00	0.6569	3	0.00	0.6569	3
47	24	25	49	9	0.00	0.5941	1	0.00	0.5941	1
47	24	25	49	9	180.00	-2.8995	2	180.00	-2.8995	2
47	24	25	49	9	0.00	0.6569	3	0.00	0.6569	3
48	25	26	50	9	0.00	0.5941	1	0.00	0.5941	1
48	25	26	50	9	180.00	-2.8995	2	180.00	-2.8995	2
48	25	26	50	9	0.00	0.6569	3	0.00	0.6569	3
48	25	26	51	9	0.00	0.5941	1	0.00	0.5941	1
48	25	26	51	9	180.00	-2.8995	2	180.00	-2.8995	2
48	25	26	51	9	0.00	0.6569	3	0.00	0.6569	3
49	25	26	50	9	0.00	0.5941	1	0.00	0.5941	1
49	25	26	50	9	180.00	-2.8995	2	180.00	-2.8995	2
49	25	26	50	9	0.00	0.6569	3	0.00	0.6569	3
49	25	26	51	9	0.00	0.5941	1	0.00	0.5941	1
49	25	26	51	9	180.00	-2.8995	2	180.00	-2.8995	2
49	25	26	51	9	0.00	0.6569	3	0.00	0.6569	3
52	31	32	54	9	0.00	0.5941	1	0.00	0.5941	1
52	31	32	54	9	180.00	-2.8995	2	180.00	-2.8995	2
52	31	32	54	9	0.00	0.6569	3	0.00	0.6569	3
52	31	32	55	9	0.00	0.5941	1	0.00	0.5941	1
52	31	32	55	9	180.00	-2.8995	2	180.00	-2.8995	2
52	31	32	55	9	0.00	0.6569	3	0.00	0.6569	3
53	31	32	54	9	0.00	0.5941	1	0.00	0.5941	1
53	31	32	54	9	180.00	-2.8995	2	180.00	-2.8995	2
53	31	32	54	9	0.00	0.6569	3	0.00	0.6569	3
53	31	32	55	9	0.00	0.5941	1	0.00	0.5941	1
53	31	32	55	9	180.00	-2.8995	2	180.00	-2.8995	2
53	31	32	55	9	0.00	0.6569	3	0.00	0.6569	3
54	32	33	56	9	0.00	0.5941	1	0.00	0.5941	1
54	32	33	56	9	180.00	-2.8995	2	180.00	-2.8995	2
54	32	33	56	9	0.00	0.6569	3	0.00	0.6569	3
54	32	33	57	9	0.00	0.5941	1	0.00	0.5941	1
54	32	33	57	9	180.00	-2.8995	2	180.00	-2.8995	2
54	32	33	57	9	0.00	0.6569	3	0.00	0.6569	3
55	32	33	56	9	0.00	0.5941	1	0.00	0.5941	1
55	32	33	56	9	180.00	-2.8995	2	180.00	-2.8995	2
55	32	33	56	9	0.00	0.6569	3	0.00	0.6569	3
55	32	33	57	9	0.00	0.5941	1	0.00	0.5941	1

```
 55  32  33  57 9 180.00  -2.8995 2   180.00  -2.8995 2
 55  32  33  57 9   0.00   0.6569 3     0.00   0.6569 3
 56  33  34  58 9   0.00   0.5941 1     0.00   0.5941 1
 56  33  34  58 9 180.00  -2.8995 2   180.00  -2.8995 2
 56  33  34  58 9   0.00   0.6569 3     0.00   0.6569 3
 56  33  34  59 9   0.00   0.5941 1     0.00   0.5941 1
 56  33  34  59 9 180.00  -2.8995 2   180.00  -2.8995 2
 56  33  34  59 9   0.00   0.6569 3     0.00   0.6569 3
 57  33  34  58 9   0.00   0.5941 1     0.00   0.5941 1
 57  33  34  58 9 180.00  -2.8995 2   180.00  -2.8995 2
 57  33  34  58 9   0.00   0.6569 3     0.00   0.6569 3
 57  33  34  59 9   0.00   0.5941 1     0.00   0.5941 1
 57  33  34  59 9 180.00  -2.8995 2   180.00  -2.8995 2
 57  33  34  59 9   0.00   0.6569 3     0.00   0.6569 3

[ dihedrals ]
; ai aj ak al fu xi0 kxi xi0 kxi
  4   3   1  37 2   0.00  27.6981     0.00  27.6981
  3   2   4  17 2   0.00  21.0790     0.00  21.0790
  5   9   1   6 2   0.00  30.1081     0.00  30.1081
  6   7   5  38 2   0.00  -8.4349     0.00  -8.4349
  7   8   6  18 2   0.00  30.1081     0.00  30.1081
  8   9   7  10 2   0.00  -6.6275     0.00  -6.6275
 10  11   8  15 2   0.00  21.0790     0.00  21.0790
 12  20  11  13 2   0.00  21.0790     0.00  21.0790
 18  16   7  39 2   0.00   0.0000     0.00   0.0000
 18  16   7  40 2   0.00   0.0000     0.00   0.0000
 13  14  12  19 2   0.00  21.0790     0.00  21.0790
 21  22  20  26 2   0.00   0.0000     0.00   0.0000
 21  26  20  42 2   0.00   0.0000     0.00   0.0000
 22  23  21  43 2   0.00   0.0000     0.00   0.0000
 22  43  21  44 2   0.00   0.0000     0.00   0.0000
 23  27  22  24 2   0.00   0.0000     0.00   0.0000
 23  24  22  45 2   0.00   0.0000     0.00   0.0000
 26  25  21  51 2   0.00   0.0000     0.00   0.0000
 26  51  21  50 2   0.00   0.0000     0.00   0.0000
 28  30  27  29 2   0.00  78.2910     0.00  78.2910
 30  31  28  34 2   0.00 -12.0416     0.00 -12.0416
 31  32  30  53 2   0.00   0.0000     0.00   0.0000
 31  53  30  52 2   0.00   0.0000     0.00   0.0000
 32  33  31  55 2   0.00   0.0000     0.00   0.0000
 32  55  31  54 2   0.00   0.0000     0.00   0.0000
  2   9   3  35 2   0.00   7.2300     0.00   7.2300
 14  13  15  41 2   0.00  27.6981     0.00  27.6981
 24  25  23  46 2   0.00   0.0000     0.00   0.0000
 24  25  23  47 2   0.00   0.0000     0.00   0.0000
 25  24  26  48 2   0.00   0.0000     0.00   0.0000
 25  48  26  49 2   0.00   0.0000     0.00   0.0000
 33  34  32  56 2   0.00   0.0000     0.00   0.0000
 33  34  32  57 2   0.00   0.0000     0.00   0.0000
 34  33  30  58 2   0.00   0.0000     0.00   0.0000
 34  58  30  59 2   0.00   0.0000     0.00   0.0000
```

3) The inhibitor (16):

```
[ atomtypes ]
; name at.num  mass   charge  ptype    sigma           epsilon
```

```
NPYD    7   14.0067  0.0  A        0.329632    0.836800
CB      6   12.0110  0.0  A        0.355005    0.292880
C5A     6   12.0110  0.0  A        0.363487    0.209200
NPYL    7   14.0067  0.0  A        0.306469    0.376560
C5B     6   12.0110  0.0  A        0.363487    0.209200
F       9   18.9984  0.0  A        0.290433    0.564840
CR      6   12.0110  0.0  A        0.387541    0.230120
NM      7   14.0067  0.0  A        0.329632    0.836800
C=O     6   12.0110  0.0  A        0.356359    0.460240
O=C     8   15.9994  0.0  A        0.302905    0.502080
NC=O    7   14.0067  0.0  A        0.329632    0.836800
HCMM    1    1.0079  0.0  A        0.235197    0.092048
HNR     1    1.0079  0.0  A        0.040001    0.192464


[ pairtypes ]
; i    j    func    sigma1-4    epsilon1-4 ; THESE ARE 1-4 INTERACTIONS
CR      NPYD  1     0.334087    0.187114
CR      CB    1     0.346773    0.110698
CR      C5A   1     0.351014    0.093557
CR      NPYL  1     0.322505    0.125520
CR      C5B   1     0.351014    0.093557
CR      F     1     0.314487    0.153730
CR      CR    1     0.338541    0.041840
CR      NM    1     0.334087    0.187114
CR      C=O   1     0.347450    0.138768
CR      O=C   1     0.293997    0.144938
CR      NC=O  1     0.334087    0.187114
CR      HCMM  1     0.286869    0.062059
CR      HNR   1     0.189271    0.089737
O=C     NPYD  1     0.289542    0.648182
O=C     CB    1     0.302228    0.383470
O=C     C5A   1     0.306469    0.324091
O=C     NPYL  1     0.277960    0.434814
O=C     C5B   1     0.306469    0.324091
O=C     F     1     0.269942    0.532536
O=C     NM    1     0.289542    0.648182
O=C     C=O   1     0.302905    0.480705
O=C     O=C   1     0.249452    0.502080
O=C     NC=O  1     0.289542    0.648182
O=C     HCMM  1     0.242324    0.214978
O=C     HNR   1     0.144726    0.310857

[ moleculetype ]
; Name nrexcl
16 3

[ atoms ]
; nr type resnr resid atom cgnr charge mass
   1 NPYD 1  LIG N       1 -0.5670  14.0067
   2 CB   1  LIG C       2 -0.1500  12.0110
   3 CB   1  LIG C1      3  0.1900  12.0110
   4 CB   1  LIG C2      4  0.1600  12.0110
   5 C5A  1  LIG C3      5  0.1054  12.0110
   6 NPYL 1  LIG N1      6  0.0332  14.0067
   7 C5A  1  LIG C4      7 -0.3316  12.0110
   8 C5B  1  LIG C6      8 -0.0540  12.0110
```

```
  9 C5B  1  LIG C7      9  0.0000  12.0110
 10 CB   1  LIG C8     10  0.6740  12.0110
 11 NPYD 1  LIG N2     11 -0.6200  14.0067
 12 CB   1  LIG C9     12  0.0580  12.0110
 13 CB   1  LIG C10    13  0.1900  12.0110
 14 CB   1  LIG C11    14  0.1600  12.0110
 15 NPYD 1  LIG N3     15 -0.6200  14.0067
 16 F    1  LIG F      16 -0.1900  18.9984
 17 CR   1  LIG C5     17  0.1800  12.0110
 18 F    1  LIG F1     18 -0.1900  18.9984
 19 NM   1  LIG N4     19 -0.6980  14.0067
 20 CR   1  LIG C12    20 -0.0500  12.0110
 21 CR   1  LIG C13    21  0.0000  12.0110
 22 CR   1  LIG C14    22 -0.0500  12.0110
 23 CR   1  LIG C15    23  0.0000  12.0110
 24 CR   1  LIG C16    24  0.0000  12.0110
 25 CR   1  LIG C17    25  0.0000  12.0110
 26 NM   1  LIG N5     26 -0.7300  14.0067
 27 C=O  1  LIG C18    27  0.4100  12.0110
 28 O=C  1  LIG O      28 -0.5700  15.9994
 29 NC=O 1  LIG N6     29 -0.6602  14.0067
 30 CR   1  LIG C19    30  0.3001  12.0110
 31 CR   1  LIG C20    31  0.0000  12.0110
 32 CR   1  LIG C21    32  0.0000  12.0110
 33 CR   1  LIG C22    33  0.3001  12.0110
 34 HCMM 1  LIG H      34  0.1500   1.0079
 35 HCMM 1  LIG H1     35  0.1500   1.0079
 36 HNR  1  LIG H2     36  0.2700   1.0079
 37 HCMM 1  LIG H3     37  0.0000   1.0079
 38 HCMM 1  LIG H4     38  0.0000   1.0079
 39 HCMM 1  LIG H5     39  0.0000   1.0079
 40 HCMM 1  LIG H6     40  0.1500   1.0079
 41 HCMM 1  LIG H7     41  0.0000   1.0079
 42 HCMM 1  LIG H8     42  0.0000   1.0079
 43 HCMM 1  LIG H9     43  0.0000   1.0079
 44 HCMM 1  LIG H10    44  0.0000   1.0079
 45 HCMM 1  LIG H11    45  0.0000   1.0079
 46 HCMM 1  LIG H12    46  0.0000   1.0079
 47 HCMM 1  LIG H13    47  0.0000   1.0079
 48 HCMM 1  LIG H14    48  0.0000   1.0079
 49 HCMM 1  LIG H15    49  0.0000   1.0079
 50 HCMM 1  LIG H16    50  0.0000   1.0079
 51 HCMM 1  LIG H17    51  0.0000   1.0079
 52 HCMM 1  LIG H18    52  0.0000   1.0079
 53 HCMM 1  LIG H19    53  0.0000   1.0079
 54 HCMM 1  LIG H20    54  0.0000   1.0079
 55 HCMM 1  LIG H21    55  0.0000   1.0079
 56 HCMM 1  LIG H22    56  0.0000   1.0079
 57 HCMM 1  LIG H23    57  0.0000   1.0079
 58 HCMM 1  LIG H24    58  0.0000   1.0079

[ bonds ]
; ai aj fu b0 kb, b0 kb
 36   6 1 0.10120  428294.3  0.10120  428294.3
 39  17 1 0.10930  287014.9  0.10930  287014.9
  6   5 1 0.13640  379454.5  0.13640  379454.5
  6   7 1 0.13640  379454.5  0.13640  379454.5
```

17	38	1	0.10930	287014.9	0.10930	287014.9
17	37	1	0.10930	287014.9	0.10930	287014.9
17	7	1	0.14710	269852.1	0.14710	269852.1
1	5	1	0.13300	439556.0	0.13300	439556.0
1	4	1	0.13330	345489.6	0.13330	345489.6
5	9	1	0.13770	428655.8	0.13770	428655.8
7	8	1	0.13770	428655.8	0.13770	428655.8
35	4	1	0.10840	319534.6	0.10840	319534.6
4	3	1	0.13740	335613.7	0.13740	335613.7
8	9	1	0.14180	259734.4	0.14180	259734.4
8	10	1	0.14320	317065.2	0.14320	317065.2
9	2	1	0.14320	317065.2	0.14320	317065.2
3	2	1	0.13740	335613.7	0.13740	335613.7
3	16	1	0.13420	392101.0	0.13420	392101.0
2	34	1	0.10840	319534.6	0.10840	319534.6
15	10	1	0.13330	345489.6	0.13330	345489.6
15	14	1	0.13330	345489.6	0.13330	345489.6
10	11	1	0.13330	345489.6	0.13330	345489.6
40	14	1	0.10840	319534.6	0.10840	319534.6
14	13	1	0.13740	335613.7	0.13740	335613.7
11	12	1	0.13330	345489.6	0.13330	345489.6
13	12	1	0.13740	335613.7	0.13740	335613.7
13	18	1	0.13420	392101.0	0.13420	392101.0
12	19	1	0.13350	429799.7	0.13350	429799.7
41	20	1	0.10930	287014.9	0.10930	287014.9
19	20	1	0.14440	268346.7	0.14440	268346.7
50	25	1	0.10930	287014.9	0.10930	287014.9
20	25	1	0.15080	256422.3	0.15080	256422.3
20	21	1	0.15080	256422.3	0.15080	256422.3
42	21	1	0.10930	287014.9	0.10930	287014.9
47	24	1	0.10930	287014.9	0.10930	287014.9
44	22	1	0.10930	287014.9	0.10930	287014.9
25	24	1	0.15080	256422.3	0.15080	256422.3
25	49	1	0.10930	287014.9	0.10930	287014.9
21	22	1	0.15080	256422.3	0.15080	256422.3
21	43	1	0.10930	287014.9	0.10930	287014.9
24	48	1	0.10930	287014.9	0.10930	287014.9
24	23	1	0.15080	256422.3	0.15080	256422.3
22	23	1	0.15080	256422.3	0.15080	256422.3
22	26	1	0.14440	268346.7	0.14440	268346.7
57	33	1	0.10930	287014.9	0.10930	287014.9
23	45	1	0.10930	287014.9	0.10930	287014.9
23	46	1	0.10930	287014.9	0.10930	287014.9
26	27	1	0.13220	455755.6	0.13220	455755.6
58	33	1	0.10930	287014.9	0.10930	287014.9
33	29	1	0.14360	280872.8	0.14360	280872.8
33	32	1	0.15080	256422.3	0.15080	256422.3
27	29	1	0.13690	351030.1	0.13690	351030.1
27	28	1	0.12220	779866.6	0.12220	779866.6
56	32	1	0.10930	287014.9	0.10930	287014.9
29	30	1	0.14360	280872.8	0.14360	280872.8
55	32	1	0.10930	287014.9	0.10930	287014.9
32	31	1	0.15080	256422.3	0.15080	256422.3
52	30	1	0.10930	287014.9	0.10930	287014.9
30	31	1	0.15080	256422.3	0.15080	256422.3
30	51	1	0.10930	287014.9	0.10930	287014.9
31	53	1	0.10930	287014.9	0.10930	287014.9

31 54 1 0.10930 287014.9 0.10930 287014.9

[pairs]
; ai aj fu
1 2 1
1 16 1
1 7 1
1 36 1
1 8 1
2 35 1
2 6 1
2 7 1
2 10 1
3 5 1
3 8 1
4 6 1
4 9 1
4 34 1
5 35 1
5 17 1
5 34 1
5 10 1
6 10 1
6 37 1
6 38 1
6 39 1
7 11 1
7 15 1
8 36 1
8 37 1
8 38 1
8 39 1
8 34 1
8 12 1
8 14 1
9 16 1
9 36 1
9 17 1
9 11 1
9 15 1
10 17 1
10 13 1
10 19 1
10 40 1
11 14 1
11 18 1
11 20 1
12 15 1
12 40 1
12 21 1
12 25 1
12 41 1
13 20 1
14 19 1
15 18 1
16 34 1
16 35 1

17	36	1
18	19	1
18	40	1
19	22	1
19	42	1
19	43	1
19	24	1
19	49	1
19	50	1
20	23	1
20	26	1
20	44	1
20	47	1
20	48	1
21	24	1
21	49	1
21	50	1
21	45	1
21	46	1
21	27	1
22	25	1
22	41	1
22	47	1
22	48	1
22	28	1
22	29	1
23	42	1
23	43	1
23	27	1
23	49	1
23	50	1
24	26	1
24	44	1
24	41	1
25	42	1
25	43	1
25	45	1
25	46	1
26	42	1
26	43	1
26	45	1
26	46	1
26	30	1
26	33	1
27	44	1
27	31	1
27	51	1
27	52	1
27	32	1
27	57	1
27	58	1
28	30	1
28	33	1
29	53	1
29	54	1
29	55	1
29	56	1

```
 30  57 1
 30  58 1
 30  55 1
 30  56 1
 31  57 1
 31  58 1
 32  51 1
 32  52 1
 33  51 1
 33  52 1
 33  53 1
 33  54 1
 41  42 1
 41  43 1
 41  49 1
 41  50 1
 42  44 1
 43  44 1
 44  45 1
 44  46 1
 45  47 1
 45  48 1
 46  47 1
 46  48 1
 47  49 1
 47  50 1
 48  49 1
 48  50 1
 51  53 1
 51  54 1
 52  53 1
 52  54 1
 53  55 1
 53  56 1
 54  55 1
 54  56 1
 55  57 1
 55  58 1
 56  57 1
 56  58 1

[ angles ]
; ai aj ak fu th0 kth ub0 kub th0 kth ub0 kub
  4   1    5 1  110.1810  740.72    110.1810  740.72
  3   2    9 1  112.5670  254.73    112.5670  254.73
  3   2   34 1   120.5710  339.05    120.5710  339.05
  9   2   34 1   121.4460  314.95    121.4460  314.95
  2   3    4 1  119.9770  402.88    119.9770  402.88
  2   3   16 1   118.0650  658.81    118.0650  658.81
  4   3   16 1   118.0650  658.81    118.0650  658.81
  1   4    3 1  126.1390  358.92    126.1390  358.92
  1   4   35 1   115.5880  417.33    115.5880  417.33
  3   4   35 1   120.5710  339.05    120.5710  339.05
  1   5    6 1  124.8140  615.46    124.8140  615.46
  1   5    9 1  126.5130  548.01    126.5130  548.01
  6   5    9 1  107.2550  489.59    107.2550  489.59
  5   6    7 1  109.5990  693.74    109.5990  693.74
```

5	6	36	1	127.7700	331.82	127.7700	331.82
7	6	36	1	127.7700	331.82	127.7700	331.82
6	7	8	1	107.2550	489.59	107.2550	489.59
6	7	17	1	121.8320	563.07	121.8320	563.07
8	7	17	1	131.3780	443.83	131.3780	443.83
7	8	9	1	108.2390	521.51	108.2390	521.51
7	8	10	1	120.0000	602.21	120.0000	602.21
9	8	10	1	128.6730	464.91	128.6730	464.91
2	9	5	1	120.0000	602.21	120.0000	602.21
2	9	8	1	128.6730	464.91	128.6730	464.91
5	9	8	1	108.2390	521.51	108.2390	521.51
8	10	11	1	128.6730	464.91	128.6730	464.91
8	10	15	1	128.6730	464.91	128.6730	464.91
11	10	15	1	128.9380	436.60	128.9380	436.60
10	11	12	1	115.4060	653.40	115.4060	653.40
11	12	13	1	126.1390	358.92	126.1390	358.92
11	12	19	1	118.3490	691.34	118.3490	691.34
13	12	19	1	124.3840	566.68	124.3840	566.68
12	13	14	1	119.9770	402.88	119.9770	402.88
12	13	18	1	118.0650	658.81	118.0650	658.81
14	13	18	1	118.0650	658.81	118.0650	658.81
13	14	15	1	126.1390	358.92	126.1390	358.92
13	14	40	1	120.5710	339.05	120.5710	339.05
15	14	40	1	115.5880	417.33	115.5880	417.33
10	15	14	1	115.4060	653.40	115.4060	653.40
7	17	37	1	110.4670	373.97	110.4670	373.97
7	17	38	1	110.4670	373.97	110.4670	373.97
7	17	39	1	110.4670	373.97	110.4670	373.97
37	17	38	1	108.8360	310.74	108.8360	310.74
37	17	39	1	108.8360	310.74	108.8360	310.74
38	17	39	1	108.8360	310.74	108.8360	310.74
12	19	20	1	120.0000	602.21	120.0000	602.21
19	20	21	1	109.5000	602.21	109.5000	602.21
19	20	25	1	109.5000	602.21	109.5000	602.21
19	20	41	1	113.0350	394.45	113.0350	394.45
21	20	25	1	109.6080	512.48	109.6080	512.48
21	20	41	1	110.5490	383.00	110.5490	383.00
25	20	41	1	110.5490	383.00	110.5490	383.00
20	21	22	1	109.6080	512.48	109.6080	512.48
20	21	42	1	110.5490	383.00	110.5490	383.00
20	21	43	1	110.5490	383.00	110.5490	383.00
22	21	42	1	110.5490	383.00	110.5490	383.00
22	21	43	1	110.5490	383.00	110.5490	383.00
42	21	43	1	108.8360	310.74	108.8360	310.74
21	22	23	1	109.6080	512.48	109.6080	512.48
21	22	26	1	109.5000	602.21	109.5000	602.21
21	22	44	1	110.5490	383.00	110.5490	383.00
23	22	26	1	109.5000	602.21	109.5000	602.21
23	22	44	1	110.5490	383.00	110.5490	383.00
26	22	44	1	113.0350	394.45	113.0350	394.45
22	23	24	1	109.6080	512.48	109.6080	512.48
22	23	45	1	110.5490	383.00	110.5490	383.00
22	23	46	1	110.5490	383.00	110.5490	383.00
24	23	45	1	110.5490	383.00	110.5490	383.00
24	23	46	1	110.5490	383.00	110.5490	383.00
45	23	46	1	108.8360	310.74	108.8360	310.74
23	24	25	1	109.6080	512.48	109.6080	512.48

```
 23  24  47 1  110.5490  383.00    110.5490  383.00
 23  24  48 1  110.5490  383.00    110.5490  383.00
 25  24  47 1  110.5490  383.00    110.5490  383.00
 25  24  48 1  110.5490  383.00    110.5490  383.00
 47  24  48 1  108.8360  310.74    108.8360  310.74
 20  25  24 1  109.6080  512.48    109.6080  512.48
 20  25  49 1  110.5490  383.00    110.5490  383.00
 20  25  50 1  110.5490  383.00    110.5490  383.00
 24  25  49 1  110.5490  383.00    110.5490  383.00
 24  25  50 1  110.5490  383.00    110.5490  383.00
 49  25  50 1  108.8360  310.74    108.8360  310.74
 22  26  27 1  120.0000  602.21    120.0000  602.21
 26  27  28 1  129.3490  679.89    129.3490  679.89
 26  27  29 1  120.0000  602.21    120.0000  602.21
 28  27  29 1  127.1520  546.20    127.1520  546.20
 27  29  30 1  119.6000  494.41    119.6000  494.41
 27  29  33 1  119.6000  494.41    119.6000  494.41
 30  29  33 1  117.9090  672.67    117.9090  672.67
 29  30  31 1  109.9600  632.32    109.9600  632.32
 29  30  51 1  107.6460  445.64    107.6460  445.64
 29  30  52 1  107.6460  445.64    107.6460  445.64
 31  30  51 1  110.5490  383.00    110.5490  383.00
 31  30  52 1  110.5490  383.00    110.5490  383.00
 51  30  52 1  108.8360  310.74    108.8360  310.74
 30  31  32 1  109.6080  512.48    109.6080  512.48
 30  31  53 1  110.5490  383.00    110.5490  383.00
 30  31  54 1  110.5490  383.00    110.5490  383.00
 32  31  53 1  110.5490  383.00    110.5490  383.00
 32  31  54 1  110.5490  383.00    110.5490  383.00
 53  31  54 1  108.8360  310.74    108.8360  310.74
 31  32  33 1  109.6080  512.48    109.6080  512.48
 31  32  55 1  110.5490  383.00    110.5490  383.00
 31  32  56 1  110.5490  383.00    110.5490  383.00
 33  32  55 1  110.5490  383.00    110.5490  383.00
 33  32  56 1  110.5490  383.00    110.5490  383.00
 55  32  56 1  108.8360  310.74    108.8360  310.74
 29  33  32 1  109.9600  632.32    109.9600  632.32
 29  33  57 1  107.6460  445.64    107.6460  445.64
 29  33  58 1  107.6460  445.64    107.6460  445.64
 32  33  57 1  110.5490  383.00    110.5490  383.00
 32  33  58 1  110.5490  383.00    110.5490  383.00
 57  33  58 1  108.8360  310.74    108.8360  310.74

[ dihedrals ]
; ai aj ak al fu phi0 kphi mult phi0 kphi mult
  1   4   3   2 9 180.00  14.6440 2   180.00  14.6440 2
  1   4   3  16 9 180.00  14.6440 2   180.00  14.6440 2
  1   5   6   7 9 180.00   8.3680 2   180.00   8.3680 2
  1   5   6  36 9 180.00   8.3680 2   180.00   8.3680 2
  1   5   9   2 9 180.00  14.6440 2   180.00  14.6440 2
  1   5   9   8 9 180.00  14.6440 2   180.00  14.6440 2
  2   3   4  35 9 180.00  14.6440 2   180.00  14.6440 2
  2   9   5   6 9 180.00  14.6440 2   180.00  14.6440 2
  2   9   8   7 9 180.00  14.6440 2   180.00  14.6440 2
  2   9   8  10 9 180.00  14.6440 2   180.00  14.6440 2
  3   2   9   5 9 180.00  14.6440 2   180.00  14.6440 2
  3   2   9   8 9 180.00  14.6440 2   180.00  14.6440 2
```

3	4	1	5	9	180.00	14.6440	2	180.00	14.6440	2
4	1	5	6	9	180.00	14.6440	2	180.00	14.6440	2
4	1	5	9	9	180.00	14.6440	2	180.00	14.6440	2
4	3	2	9	9	180.00	14.6440	2	180.00	14.6440	2
4	3	2	34	9	180.00	14.6440	2	180.00	14.6440	2
5	1	4	35	9	180.00	14.6440	2	180.00	14.6440	2
5	6	7	8	9	180.00	8.3680	2	180.00	8.3680	2
5	6	7	17	9	180.00	8.3680	2	180.00	8.3680	2
5	9	2	34	9	180.00	14.6440	2	180.00	14.6440	2
5	9	8	7	9	180.00	14.6440	2	180.00	14.6440	2
5	9	8	10	9	180.00	14.6440	2	180.00	14.6440	2
6	5	9	8	9	180.00	14.6440	2	180.00	14.6440	2
6	7	8	9	9	180.00	14.6440	2	180.00	14.6440	2
6	7	8	10	9	180.00	14.6440	2	180.00	14.6440	2
7	6	5	9	9	180.00	8.3680	2	180.00	8.3680	2
7	8	10	11	9	180.00	3.7656	2	180.00	3.7656	2
7	8	10	15	9	180.00	3.7656	2	180.00	3.7656	2
8	7	6	36	9	180.00	8.3680	2	180.00	8.3680	2
8	9	2	34	9	180.00	14.6440	2	180.00	14.6440	2
9	2	3	16	9	180.00	14.6440	2	180.00	14.6440	2
9	5	6	36	9	180.00	8.3680	2	180.00	8.3680	2
9	8	7	17	9	180.00	14.6440	2	180.00	14.6440	2
9	8	10	11	9	180.00	3.7656	2	180.00	3.7656	2
9	8	10	15	9	180.00	3.7656	2	180.00	3.7656	2
10	11	12	13	9	180.00	14.6440	2	180.00	14.6440	2
10	11	12	19	9	180.00	14.6440	2	180.00	14.6440	2
10	15	14	13	9	180.00	14.6440	2	180.00	14.6440	2
10	15	14	40	9	180.00	14.6440	2	180.00	14.6440	2
11	10	15	14	9	180.00	14.6440	2	180.00	14.6440	2
11	12	13	14	9	180.00	14.6440	2	180.00	14.6440	2
11	12	13	18	9	180.00	14.6440	2	180.00	14.6440	2
11	12	19	20	9	180.00	7.5312	2	180.00	7.5312	2
12	11	10	15	9	180.00	14.6440	2	180.00	14.6440	2
12	13	14	15	9	180.00	14.6440	2	180.00	14.6440	2
12	13	14	40	9	180.00	14.6440	2	180.00	14.6440	2
12	19	20	21	9	0.00	0.5230	3	0.00	0.5230	3
12	19	20	25	9	0.00	0.5230	3	0.00	0.5230	3
12	19	20	41	9	0.00	0.5230	3	0.00	0.5230	3
13	12	19	20	9	180.00	7.5312	2	180.00	7.5312	2
14	13	12	19	9	180.00	14.6440	2	180.00	14.6440	2
15	14	13	18	9	180.00	14.6440	2	180.00	14.6440	2
16	3	2	34	9	180.00	14.6440	2	180.00	14.6440	2
16	3	4	35	9	180.00	14.6440	2	180.00	14.6440	2
17	7	6	36	9	180.00	8.3680	2	180.00	8.3680	2
18	13	12	19	9	180.00	14.6440	2	180.00	14.6440	2
18	13	14	40	9	180.00	14.6440	2	180.00	14.6440	2
19	20	21	22	9	0.00	0.6276	3	0.00	0.6276	3
19	20	21	42	9	0.00	0.6276	3	0.00	0.6276	3
19	20	21	43	9	0.00	0.6276	3	0.00	0.6276	3
19	20	25	24	9	0.00	0.6276	3	0.00	0.6276	3
19	20	25	49	9	0.00	0.6276	3	0.00	0.6276	3
19	20	25	50	9	0.00	0.6276	3	0.00	0.6276	3
20	21	22	23	9	0.00	0.2134	1	0.00	0.2134	1
20	21	22	23	9	180.00	1.4267	2	180.00	1.4267	2
20	21	22	23	9	0.00	0.6945	3	0.00	0.6945	3
20	21	22	26	9	0.00	0.6276	3	0.00	0.6276	3
20	21	22	44	9	0.00	1.3389	1	0.00	1.3389	1

20	21	22	44	9	180.00	-1.3180	2	180.00	-1.3180	2
20	21	22	44	9	0.00	0.5523	3	0.00	0.5523	3
20	25	24	23	9	0.00	0.2134	1	0.00	0.2134	1
20	25	24	23	9	180.00	1.4267	2	180.00	1.4267	2
20	25	24	23	9	0.00	0.6945	3	0.00	0.6945	3
20	25	24	47	9	0.00	1.3389	1	0.00	1.3389	1
20	25	24	47	9	180.00	-1.3180	2	180.00	-1.3180	2
20	25	24	47	9	0.00	0.5523	3	0.00	0.5523	3
20	25	24	48	9	0.00	1.3389	1	0.00	1.3389	1
20	25	24	48	9	180.00	-1.3180	2	180.00	-1.3180	2
20	25	24	48	9	0.00	0.5523	3	0.00	0.5523	3
21	20	25	24	9	0.00	0.2134	1	0.00	0.2134	1
21	20	25	24	9	180.00	1.4267	2	180.00	1.4267	2
21	20	25	24	9	0.00	0.6945	3	0.00	0.6945	3
21	20	25	49	9	0.00	1.3389	1	0.00	1.3389	1
21	20	25	49	9	180.00	-1.3180	2	180.00	-1.3180	2
21	20	25	49	9	0.00	0.5523	3	0.00	0.5523	3
21	20	25	50	9	0.00	1.3389	1	0.00	1.3389	1
21	20	25	50	9	180.00	-1.3180	2	180.00	-1.3180	2
21	20	25	50	9	0.00	0.5523	3	0.00	0.5523	3
21	22	23	24	9	0.00	0.2134	1	0.00	0.2134	1
21	22	23	24	9	180.00	1.4267	2	180.00	1.4267	2
21	22	23	24	9	0.00	0.6945	3	0.00	0.6945	3
21	22	23	45	9	0.00	1.3389	1	0.00	1.3389	1
21	22	23	45	9	180.00	-1.3180	2	180.00	-1.3180	2
21	22	23	45	9	0.00	0.5523	3	0.00	0.5523	3
21	22	23	46	9	0.00	1.3389	1	0.00	1.3389	1
21	22	23	46	9	180.00	-1.3180	2	180.00	-1.3180	2
21	22	23	46	9	0.00	0.5523	3	0.00	0.5523	3
21	22	26	27	9	0.00	0.5230	3	0.00	0.5230	3
22	21	20	25	9	0.00	0.2134	1	0.00	0.2134	1
22	21	20	25	9	180.00	1.4267	2	180.00	1.4267	2
22	21	20	25	9	0.00	0.6945	3	0.00	0.6945	3
22	21	20	41	9	0.00	1.3389	1	0.00	1.3389	1
22	21	20	41	9	180.00	-1.3180	2	180.00	-1.3180	2
22	21	20	41	9	0.00	0.5523	3	0.00	0.5523	3
22	23	24	25	9	0.00	0.2134	1	0.00	0.2134	1
22	23	24	25	9	180.00	1.4267	2	180.00	1.4267	2
22	23	24	25	9	0.00	0.6945	3	0.00	0.6945	3
22	23	24	47	9	0.00	1.3389	1	0.00	1.3389	1
22	23	24	47	9	180.00	-1.3180	2	180.00	-1.3180	2
22	23	24	47	9	0.00	0.5523	3	0.00	0.5523	3
22	23	24	48	9	0.00	1.3389	1	0.00	1.3389	1
22	23	24	48	9	180.00	-1.3180	2	180.00	-1.3180	2
22	23	24	48	9	0.00	0.5523	3	0.00	0.5523	3
22	26	27	28	9	180.00	7.5312	2	180.00	7.5312	2
22	26	27	29	9	180.00	7.5312	2	180.00	7.5312	2
23	22	21	42	9	0.00	1.3389	1	0.00	1.3389	1
23	22	21	42	9	180.00	-1.3180	2	180.00	-1.3180	2
23	22	21	42	9	0.00	0.5523	3	0.00	0.5523	3
23	22	21	43	9	0.00	1.3389	1	0.00	1.3389	1
23	22	21	43	9	180.00	-1.3180	2	180.00	-1.3180	2
23	22	21	43	9	0.00	0.5523	3	0.00	0.5523	3
23	22	26	27	9	0.00	0.5230	3	0.00	0.5230	3
23	24	25	49	9	0.00	1.3389	1	0.00	1.3389	1
23	24	25	49	9	180.00	-1.3180	2	180.00	-1.3180	2
23	24	25	49	9	0.00	0.5523	3	0.00	0.5523	3

23	24	25	50	9	0.00	1.3389	1	0.00	1.3389	1
23	24	25	50	9	180.00	-1.3180	2	180.00	-1.3180	2
23	24	25	50	9	0.00	0.5523	3	0.00	0.5523	3
24	23	22	26	9	0.00	0.6276	3	0.00	0.6276	3
24	23	22	44	9	0.00	1.3389	1	0.00	1.3389	1
24	23	22	44	9	180.00	-1.3180	2	180.00	-1.3180	2
24	23	22	44	9	0.00	0.5523	3	0.00	0.5523	3
24	25	20	41	9	0.00	1.3389	1	0.00	1.3389	1
24	25	20	41	9	180.00	-1.3180	2	180.00	-1.3180	2
24	25	20	41	9	0.00	0.5523	3	0.00	0.5523	3
25	20	21	42	9	0.00	1.3389	1	0.00	1.3389	1
25	20	21	42	9	180.00	-1.3180	2	180.00	-1.3180	2
25	20	21	42	9	0.00	0.5523	3	0.00	0.5523	3
25	20	21	43	9	0.00	1.3389	1	0.00	1.3389	1
25	20	21	43	9	180.00	-1.3180	2	180.00	-1.3180	2
25	20	21	43	9	0.00	0.5523	3	0.00	0.5523	3
25	24	23	45	9	0.00	1.3389	1	0.00	1.3389	1
25	24	23	45	9	180.00	-1.3180	2	180.00	-1.3180	2
25	24	23	45	9	0.00	0.5523	3	0.00	0.5523	3
25	24	23	46	9	0.00	1.3389	1	0.00	1.3389	1
25	24	23	46	9	180.00	-1.3180	2	180.00	-1.3180	2
25	24	23	46	9	0.00	0.5523	3	0.00	0.5523	3
26	22	21	42	9	0.00	0.6276	3	0.00	0.6276	3
26	22	21	43	9	0.00	0.6276	3	0.00	0.6276	3
26	22	23	45	9	0.00	0.6276	3	0.00	0.6276	3
26	22	23	46	9	0.00	0.6276	3	0.00	0.6276	3
26	27	29	30	9	180.00	12.5520	2	180.00	12.5520	2
26	27	29	33	9	180.00	12.5520	2	180.00	12.5520	2
27	26	22	44	9	0.00	0.5230	3	0.00	0.5230	3
27	29	30	31	9	0.00	-2.1464	1	0.00	-2.1464	1
27	29	30	31	9	180.00	1.4518	2	180.00	1.4518	2
27	29	30	31	9	0.00	1.9832	3	0.00	1.9832	3
27	29	30	51	9	0.00	-4.3932	1	0.00	-4.3932	1
27	29	30	51	9	180.00	2.8493	2	180.00	2.8493	2
27	29	30	51	9	0.00	0.0460	3	0.00	0.0460	3
27	29	30	52	9	0.00	-4.3932	1	0.00	-4.3932	1
27	29	30	52	9	180.00	2.8493	2	180.00	2.8493	2
27	29	30	52	9	0.00	0.0460	3	0.00	0.0460	3
27	29	33	32	9	0.00	-2.1464	1	0.00	-2.1464	1
27	29	33	32	9	180.00	1.4518	2	180.00	1.4518	2
27	29	33	32	9	0.00	1.9832	3	0.00	1.9832	3
27	29	33	57	9	0.00	-4.3932	1	0.00	-4.3932	1
27	29	33	57	9	180.00	2.8493	2	180.00	2.8493	2
27	29	33	57	9	0.00	0.0460	3	0.00	0.0460	3
27	29	33	58	9	0.00	-4.3932	1	0.00	-4.3932	1
27	29	33	58	9	180.00	2.8493	2	180.00	2.8493	2
27	29	33	58	9	0.00	0.0460	3	0.00	0.0460	3
28	27	29	30	9	0.00	-0.6694	1	0.00	-0.6694	1
28	27	29	30	9	180.00	13.1670	2	180.00	13.1670	2
28	27	29	30	9	0.00	-0.3054	3	0.00	-0.3054	3
28	27	29	33	9	0.00	-0.6694	1	0.00	-0.6694	1
28	27	29	33	9	180.00	13.1670	2	180.00	13.1670	2
28	27	29	33	9	0.00	-0.3054	3	0.00	-0.3054	3
29	30	31	32	9	0.00	0.6276	3	0.00	0.6276	3
29	30	31	53	9	0.00	0.8912	3	0.00	0.8912	3
29	30	31	54	9	0.00	0.8912	3	0.00	0.8912	3
29	33	32	31	9	0.00	0.6276	3	0.00	0.6276	3

29	33	32	55	9	0.00	0.8912	3	0.00	0.8912	3
29	33	32	56	9	0.00	0.8912	3	0.00	0.8912	3
30	29	33	32	9	0.00	0.6276	3	0.00	0.6276	3
30	29	33	57	9	0.00	1.6318	3	0.00	1.6318	3
30	29	33	58	9	0.00	1.6318	3	0.00	1.6318	3
30	31	32	33	9	0.00	0.2134	1	0.00	0.2134	1
30	31	32	33	9	180.00	1.4267	2	180.00	1.4267	2
30	31	32	33	9	0.00	0.6945	3	0.00	0.6945	3
30	31	32	55	9	0.00	1.3389	1	0.00	1.3389	1
30	31	32	55	9	180.00	-1.3180	2	180.00	-1.3180	2
30	31	32	55	9	0.00	0.5523	3	0.00	0.5523	3
30	31	32	56	9	0.00	1.3389	1	0.00	1.3389	1
30	31	32	56	9	180.00	-1.3180	2	180.00	-1.3180	2
30	31	32	56	9	0.00	0.5523	3	0.00	0.5523	3
31	30	29	33	9	0.00	0.6276	3	0.00	0.6276	3
31	32	33	57	9	0.00	1.3389	1	0.00	1.3389	1
31	32	33	57	9	180.00	-1.3180	2	180.00	-1.3180	2
31	32	33	57	9	0.00	0.5523	3	0.00	0.5523	3
31	32	33	58	9	0.00	1.3389	1	0.00	1.3389	1
31	32	33	58	9	180.00	-1.3180	2	180.00	-1.3180	2
31	32	33	58	9	0.00	0.5523	3	0.00	0.5523	3
32	31	30	51	9	0.00	1.3389	1	0.00	1.3389	1
32	31	30	51	9	180.00	-1.3180	2	180.00	-1.3180	2
32	31	30	51	9	0.00	0.5523	3	0.00	0.5523	3
32	31	30	52	9	0.00	1.3389	1	0.00	1.3389	1
32	31	30	52	9	180.00	-1.3180	2	180.00	-1.3180	2
32	31	30	52	9	0.00	0.5523	3	0.00	0.5523	3
33	29	30	51	9	0.00	1.6318	3	0.00	1.6318	3
33	29	30	52	9	0.00	1.6318	3	0.00	1.6318	3
33	32	31	53	9	0.00	1.3389	1	0.00	1.3389	1
33	32	31	53	9	180.00	-1.3180	2	180.00	-1.3180	2
33	32	31	53	9	0.00	0.5523	3	0.00	0.5523	3
33	32	31	54	9	0.00	1.3389	1	0.00	1.3389	1
33	32	31	54	9	180.00	-1.3180	2	180.00	-1.3180	2
33	32	31	54	9	0.00	0.5523	3	0.00	0.5523	3
41	20	21	42	9	0.00	0.5941	1	0.00	0.5941	1
41	20	21	42	9	180.00	-2.8995	2	180.00	-2.8995	2
41	20	21	42	9	0.00	0.6569	3	0.00	0.6569	3
41	20	21	43	9	0.00	0.5941	1	0.00	0.5941	1
41	20	21	43	9	180.00	-2.8995	2	180.00	-2.8995	2
41	20	21	43	9	0.00	0.6569	3	0.00	0.6569	3
41	20	25	49	9	0.00	0.5941	1	0.00	0.5941	1
41	20	25	49	9	180.00	-2.8995	2	180.00	-2.8995	2
41	20	25	49	9	0.00	0.6569	3	0.00	0.6569	3
41	20	25	50	9	0.00	0.5941	1	0.00	0.5941	1
41	20	25	50	9	180.00	-2.8995	2	180.00	-2.8995	2
41	20	25	50	9	0.00	0.6569	3	0.00	0.6569	3
42	21	22	44	9	0.00	0.5941	1	0.00	0.5941	1
42	21	22	44	9	180.00	-2.8995	2	180.00	-2.8995	2
42	21	22	44	9	0.00	0.6569	3	0.00	0.6569	3
43	21	22	44	9	0.00	0.5941	1	0.00	0.5941	1
43	21	22	44	9	180.00	-2.8995	2	180.00	-2.8995	2
43	21	22	44	9	0.00	0.6569	3	0.00	0.6569	3
44	22	23	45	9	0.00	0.5941	1	0.00	0.5941	1
44	22	23	45	9	180.00	-2.8995	2	180.00	-2.8995	2
44	22	23	45	9	0.00	0.6569	3	0.00	0.6569	3
44	22	23	46	9	0.00	0.5941	1	0.00	0.5941	1

44	22	23	46	9	180.00	-2.8995	2	180.00	-2.8995	2
44	22	23	46	9	0.00	0.6569	3	0.00	0.6569	3
45	23	24	47	9	0.00	0.5941	1	0.00	0.5941	1
45	23	24	47	9	180.00	-2.8995	2	180.00	-2.8995	2
45	23	24	47	9	0.00	0.6569	3	0.00	0.6569	3
45	23	24	48	9	0.00	0.5941	1	0.00	0.5941	1
45	23	24	48	9	180.00	-2.8995	2	180.00	-2.8995	2
45	23	24	48	9	0.00	0.6569	3	0.00	0.6569	3
46	23	24	47	9	0.00	0.5941	1	0.00	0.5941	1
46	23	24	47	9	180.00	-2.8995	2	180.00	-2.8995	2
46	23	24	47	9	0.00	0.6569	3	0.00	0.6569	3
46	23	24	48	9	0.00	0.5941	1	0.00	0.5941	1
46	23	24	48	9	180.00	-2.8995	2	180.00	-2.8995	2
46	23	24	48	9	0.00	0.6569	3	0.00	0.6569	3
47	24	25	49	9	0.00	0.5941	1	0.00	0.5941	1
47	24	25	49	9	180.00	-2.8995	2	180.00	-2.8995	2
47	24	25	49	9	0.00	0.6569	3	0.00	0.6569	3
47	24	25	50	9	0.00	0.5941	1	0.00	0.5941	1
47	24	25	50	9	180.00	-2.8995	2	180.00	-2.8995	2
47	24	25	50	9	0.00	0.6569	3	0.00	0.6569	3
48	24	25	49	9	0.00	0.5941	1	0.00	0.5941	1
48	24	25	49	9	180.00	-2.8995	2	180.00	-2.8995	2
48	24	25	49	9	0.00	0.6569	3	0.00	0.6569	3
48	24	25	50	9	0.00	0.5941	1	0.00	0.5941	1
48	24	25	50	9	180.00	-2.8995	2	180.00	-2.8995	2
48	24	25	50	9	0.00	0.6569	3	0.00	0.6569	3
51	30	31	53	9	0.00	0.5941	1	0.00	0.5941	1
51	30	31	53	9	180.00	-2.8995	2	180.00	-2.8995	2
51	30	31	53	9	0.00	0.6569	3	0.00	0.6569	3
51	30	31	54	9	0.00	0.5941	1	0.00	0.5941	1
51	30	31	54	9	180.00	-2.8995	2	180.00	-2.8995	2
51	30	31	54	9	0.00	0.6569	3	0.00	0.6569	3
52	30	31	53	9	0.00	0.5941	1	0.00	0.5941	1
52	30	31	53	9	180.00	-2.8995	2	180.00	-2.8995	2
52	30	31	53	9	0.00	0.6569	3	0.00	0.6569	3
52	30	31	54	9	0.00	0.5941	1	0.00	0.5941	1
52	30	31	54	9	180.00	-2.8995	2	180.00	-2.8995	2
52	30	31	54	9	0.00	0.6569	3	0.00	0.6569	3
53	31	32	55	9	0.00	0.5941	1	0.00	0.5941	1
53	31	32	55	9	180.00	-2.8995	2	180.00	-2.8995	2
53	31	32	55	9	0.00	0.6569	3	0.00	0.6569	3
53	31	32	56	9	0.00	0.5941	1	0.00	0.5941	1
53	31	32	56	9	180.00	-2.8995	2	180.00	-2.8995	2
53	31	32	56	9	0.00	0.6569	3	0.00	0.6569	3
54	31	32	55	9	0.00	0.5941	1	0.00	0.5941	1
54	31	32	55	9	180.00	-2.8995	2	180.00	-2.8995	2
54	31	32	55	9	0.00	0.6569	3	0.00	0.6569	3
54	31	32	56	9	0.00	0.5941	1	0.00	0.5941	1
54	31	32	56	9	180.00	-2.8995	2	180.00	-2.8995	2
54	31	32	56	9	0.00	0.6569	3	0.00	0.6569	3
55	32	33	57	9	0.00	0.5941	1	0.00	0.5941	1
55	32	33	57	9	180.00	-2.8995	2	180.00	-2.8995	2
55	32	33	57	9	0.00	0.6569	3	0.00	0.6569	3
55	32	33	58	9	0.00	0.5941	1	0.00	0.5941	1
55	32	33	58	9	180.00	-2.8995	2	180.00	-2.8995	2
55	32	33	58	9	0.00	0.6569	3	0.00	0.6569	3
56	32	33	57	9	0.00	0.5941	1	0.00	0.5941	1

```
 56  32  33  57 9 180.00  -2.8995 2   180.00  -2.8995 2
 56  32  33  57 9   0.00   0.6569 3     0.00   0.6569 3
 56  32  33  58 9   0.00   0.5941 1     0.00   0.5941 1
 56  32  33  58 9 180.00  -2.8995 2   180.00  -2.8995 2
 56  32  33  58 9   0.00   0.6569 3     0.00   0.6569 3

[ dihedrals ]
; ai aj ak al fu xi0 kxi xi0 kxi
  4   3   1  35 2   0.00  27.6981     0.00  27.6981
  3   2   4  16 2   0.00  21.0790     0.00  21.0790
  5   9   1   6 2   0.00  30.1081     0.00  30.1081
  6   7   5  36 2   0.00  -8.4349     0.00  -8.4349
  7   8   6  17 2   0.00  30.1081     0.00  30.1081
  8   9   7  10 2   0.00  -6.6275     0.00  -6.6275
 10  11   8  15 2   0.00  21.0790     0.00  21.0790
 12  19  11  13 2   0.00  21.0790     0.00  21.0790
 13  14  12  18 2   0.00  21.0790     0.00  21.0790
 20  21  19  25 2   0.00   0.0000     0.00   0.0000
 20  25  19  41 2   0.00   0.0000     0.00   0.0000
 21  22  20  42 2   0.00   0.0000     0.00   0.0000
 21  42  20  43 2   0.00   0.0000     0.00   0.0000
 22  26  21  23 2   0.00   0.0000     0.00   0.0000
 22  23  21  44 2   0.00   0.0000     0.00   0.0000
 25  24  20  50 2   0.00   0.0000     0.00   0.0000
 25  50  20  49 2   0.00   0.0000     0.00   0.0000
 27  29  26  28 2   0.00  78.2910     0.00  78.2910
 29  33  27  30 2   0.00 -12.0416     0.00 -12.0416
 30  31  29  52 2   0.00   0.0000     0.00   0.0000
 30  52  29  51 2   0.00   0.0000     0.00   0.0000
 33  32  29  57 2   0.00   0.0000     0.00   0.0000
 33  57  29  58 2   0.00   0.0000     0.00   0.0000
  2   9   3  34 2   0.00   7.2300     0.00   7.2300
 17  39   7  38 2   0.00   0.0000     0.00   0.0000
 17  39   7  37 2   0.00   0.0000     0.00   0.0000
 14  13  15  40 2   0.00  27.6981     0.00  27.6981
 23  24  22  45 2   0.00   0.0000     0.00   0.0000
 23  24  22  46 2   0.00   0.0000     0.00   0.0000
 24  23  25  47 2   0.00   0.0000     0.00   0.0000
 24  47  25  48 2   0.00   0.0000     0.00   0.0000
 31  32  30  53 2   0.00   0.0000     0.00   0.0000
 31  32  30  54 2   0.00   0.0000     0.00   0.0000
 32  31  33  56 2   0.00   0.0000     0.00   0.0000
 32  56  33  55 2   0.00   0.0000     0.00   0.0000
```
